# Mind the gap in kidney care: translating what we know into what we do

**DOI:** 10.1590/2175-8239-JBN-2024-E007en

**Published:** 2024-07-05

**Authors:** Valerie A. Luyckx, Katherine R. Tuttle, Dina Abdellatif, Ricardo Correa-Rotter, Winston W.S. Fung, Agnès Haris, Li-Li Hsiao, Makram Khalife, Latha A. Kumaraswami, Fiona Loud, Vasundhara Raghavan, Stefanos Roumeliotis, Marianella Sierra, Ifeoma Ulasi, Bill Wang, Siu-Fai Lui, Vassilios Liakopoulos, Alessandro Balducci, The World Kidney Day Joint Steering Committee

**Affiliations:** 1 University of Zurich, Epidemiology, Biostatistics and Prevention Institute, Department of Public and Global Health, Zurich, Switzerland.; 2 Harvard Medical School, Brigham and Women’s Hospital, Department of Medicine, Renal Division, Boston, Massachusetts, USA.; 3 University of Cape Town, Department of Paediatrics and Child Health, Cape Town, South Africa.; 4 Providence Inland Northwest Health, Providence Medical Research Center, Spokane, Washington, USA.; 5 University of Washington, Department of Medicine, Nephrology Division, Seattle, Washington, USA.; 6 Cairo University Hospital, Department of Nephrology, Cairo, Egypt.; 7 National Medical Science and Nutrition Institute Salvador Zubiran, Department of Nephrology and Mineral Metabolism, Mexico City, Mexico.; 8 University of Hong Kong, Prince of Wales Hospital, Department of Medicine and Therapeutics, The Chinese Shatin, Hong Kong, China.; 9 Péterfy Hospital, Nephrology Department, Budapest, Hungary.; 10 ISN Patient Liaison Advisory Group, Brussel, Belgium.; 11 Tamilnad Kidney Research (TANKER) Foundation, Chennai, India.; 12 Aristotle University of Thessaloniki, AHEPA University Hospital Medical School, 2nd Department of Nephrology, Thessaloniki, Greece.; 13 University of Nigeria, College of Medicine, Department of Medicine, Ituku-Ozalla, Enugu, Nigeria.; 14 The Chinese University of Hong Kong, Jockey Club School of Public Health and Primary Care, Division of Health System, Policy and Management, Hong Kong, China.; 15 Italian Kidney Foundation, Rome, Italy.

**Keywords:** Chronic Kidney Disease, Equity, Kidney Care, Public Health, World Kidney Day

## Abstract

Historically, it takes an average of 17 years for new treatments to move from clinical evidence to daily practice. Given the highly effective treatments now available to prevent or delay kidney disease onset and progression, this is far too long. Now is the time to narrow the gap between what we know and what we do. Clear guidelines exist for the prevention and management of common risk factors for kidney disease, such as hypertension and diabetes, but only a fraction of people with these conditions are diagnosed worldwide, and even fewer are treated to target. Similarly, the vast majority of people living with kidney disease are unaware of their condition, because it is often silent in the early stages. Even among patients who have been diagnosed, many do not receive appropriate treatment for kidney disease. Considering the serious consequences of kidney disease progression, kidney failure, or death, it is imperative that treatments are initiated early and appropriately. Opportunities to diagnose and treat kidney disease early must be maximized beginning at the primary care level. Many systematic barriers exist, ranging from the patient to the clinician to the health systems to societal factors. To preserve and improve kidney health for everyone everywhere, each of these barriers must be acknowledged so that sustainable solutions are developed and implemented without further delay.

At least 1 in 10 people worldwide live with kidney disease^1^. According to the Global Burden of Disease study, in 2019, more than 3.1 million deaths were attributed to kidney dysfunction in 2019, making it the seventh leading risk factor for death worldwide (Figure 1 and Figure S1)^2^. However, global mortality from all kidney diseases may actually be between 5 and 11 million per year if the estimated lives lost, especially in lower-resource settings, from acute kidney injury and from lack of access to kidney replacement therapy for kidney failure (KF) are also counted^3^. These high global death rates reflect disparities in prevention, early detection, diagnosis, and treatment of chronic kidney disease (CKD)^4^. Death rates from CKD are especially high in some regions, particularly in Central Latin America and Oceania (South Pacific Islands), indicating the urgent need for action^5^.

**Figure 1:**
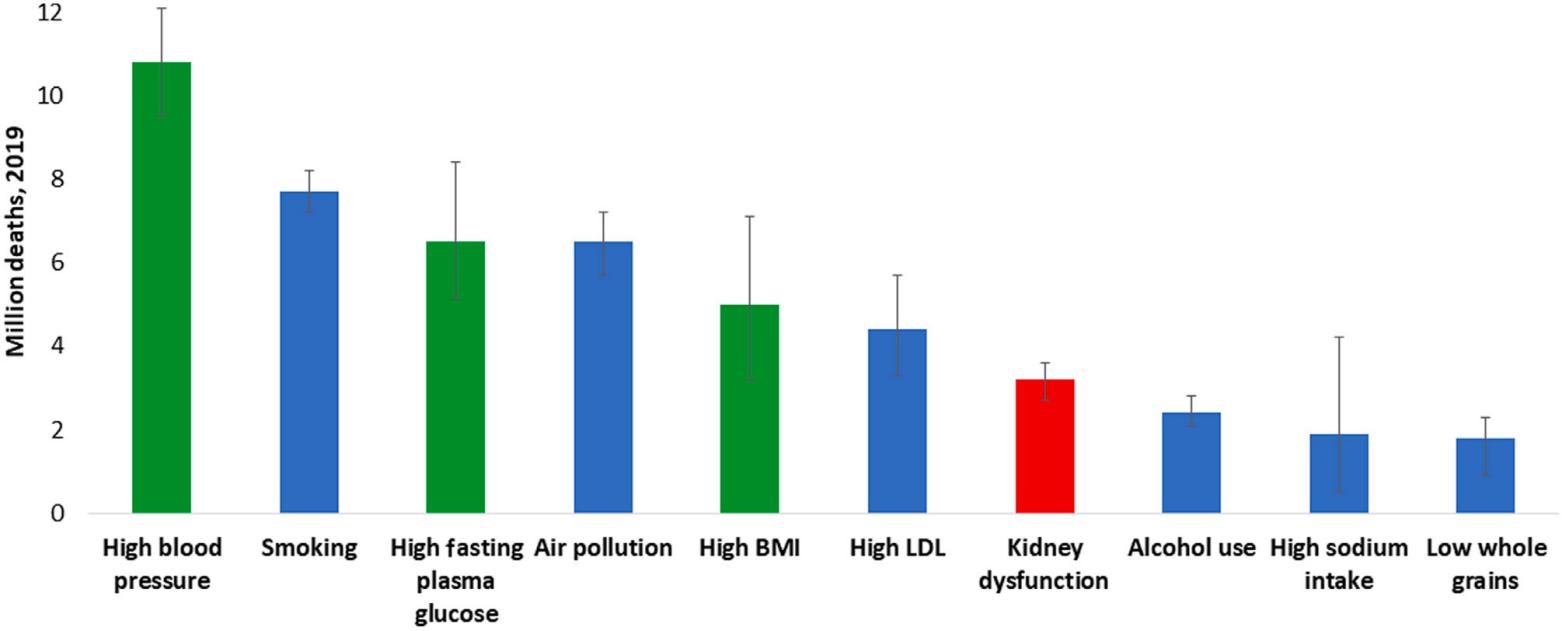
All ages, top 10 global risk factors for death, 2019. Kidney dysfunction (defined as estimated glomerular filtration rate <60 mL/min per 1.73 m^2^ or albumin-to-creatinine ratio ≥30 mg/g) was the seventh leading global level 3 risk factor for death in 2019. The 3 leading global risk factors for kidney disease, including hypertension, diabetes, and overweight/obesity, are also leading global risk factors for death; therefore, holistic strategies are required to address all risk factors simultaneously. Ranking is depicted by millions if deaths are attributed to the risk factors. Error bars depict the confidence range. Global ranking of kidney dysfunction stratified by World Bank income category and gender is shown in Supplementary Figure S1. Data obtained from the Global Burden of Disease Study^2^. Abbreviations – BMI: body mass index; LDL: low-density lipoprotein.

CKD also poses a significant global economic burden, with costs increasing exponentially as CKD progresses, not only because of dialysis and transplantation costs, but also because of the multiple comorbidities and complications that accumulate over time^6,7^. In the United States, Medicare fee-for-service spending for all beneficiaries with CKD was $86.1 billion in 2021 (22.6% of the total expenditure)^8^. Data from many resource-poor settings are absent, where most costs are paid out of pocket. A recent study from Vietnam reported that the cost of CKD per patient was higher than the gross domestic product per capita^7^. In Australia, it has been estimated that early diagnosis and prevention of CKD could save the health system 10.2 billion dollars over 20 years^9^.

Although there is regional variation in the causes of CKD, the risk factors with the highest population-attributable factors for age-standardized CKD-related disease-adjusted life years were high blood pressure (51.4%), high fasting plasma glucose level (30.9%), and high body mass index (26.5%)^10^. These risk factors are also global leading risk factors for death (Figure 1). Only 40% and 60% of those with hypertension and diabetes, respectively, are aware of their diagnosis, and a much lower proportion receive treatment and at target goals^11,12^. Moreover, at least 1 in 5 people with hypertension and 1 in 3 people with diabetes also have CKD^13^.

**Figure 2:**
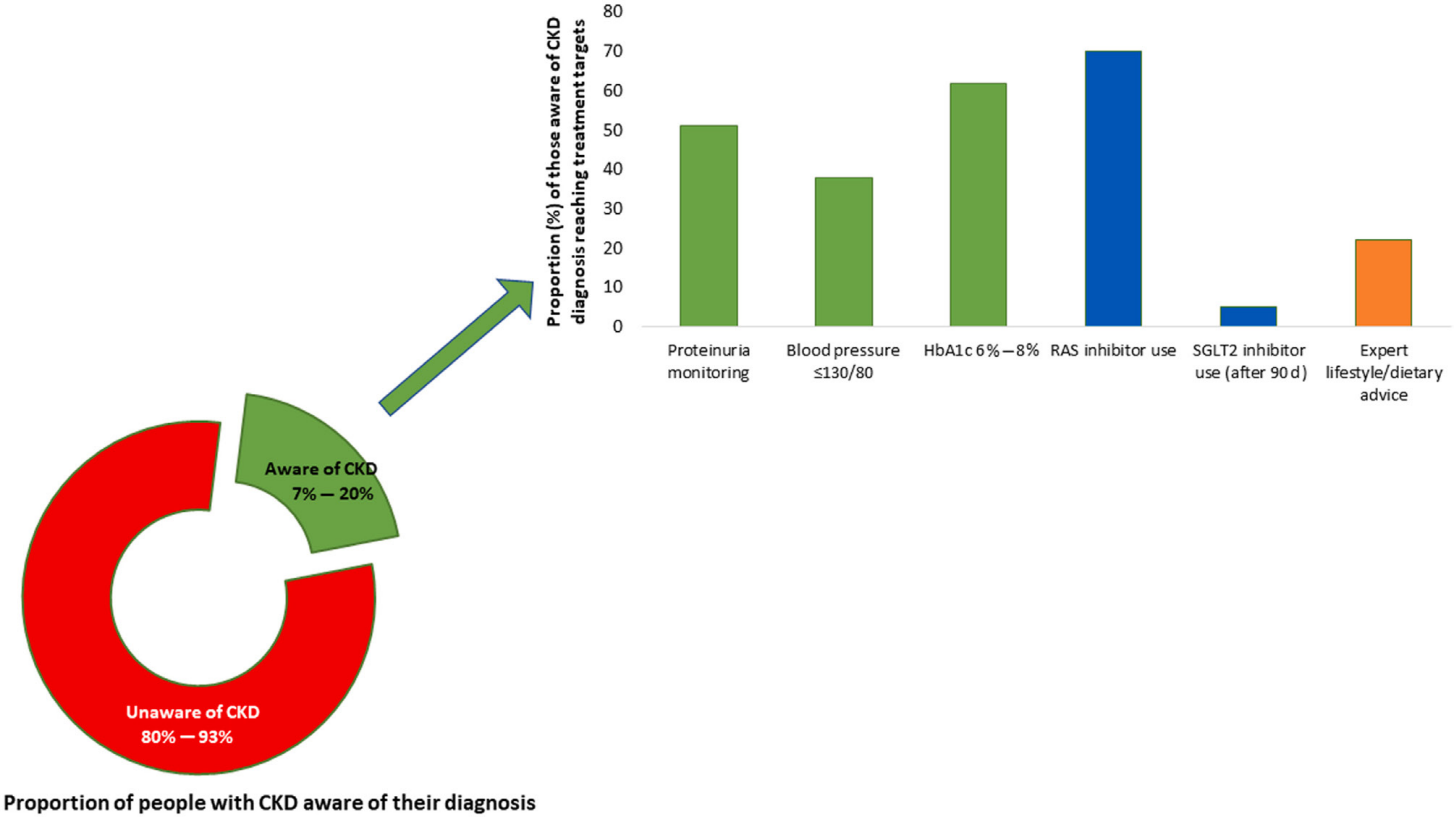
Proportion of people with chronic kidney disease (CKD) who are aware of their diagnosis and are receiving appropriate guideline-recommended care. The proportion of people with CKD who are aware of their diagnosis varies globally, with rates ranging from 7% to 20%. As CKD stage worsens, knowledge of CKD increases. Among those with a diagnosis of CKD, the average proportion of patients receiving appropriate medication to delay CKD progression (renin-angiotensin-aldosterone system [RAS] inhibitors and sodium-glucose cotransporter 2 [SGLT2] inhibitors) is suboptimal as are those reaching target blood pressure, diabetes control, and nutrition advice. The treatment targets depicted in the figure follow the Kidney Disease: Improving Global Outcomes (KDIGO) 2012 guidelines^15^. Most data come from resource-high settings; these proportions are likely lower in resource-low settings. Data are shown for proportions of patients reaching blood pressure of <130/80 mm Hg. Data compiled from previous studies^15-20^. Abbreviation – HbA1c: hemoglobin A1c.

A large proportion of CKD can be prevented through healthy lifestyles, prevention and control of risk factors, prevention of acute kidney injury, optimization of maternal and child health, mitigation of climate change, and addressing social and structural determinants of health^3^. Nevertheless, the benefits of some of these measures may only be seen in future generations. In the meantime, early diagnosis and risk stratification create opportunities to institute therapies that slow, halt, or even reverse CKD^14^. Concerningly, CKD awareness was strikingly low among individuals with kidney dysfunction, with ≈80% to 95% of patients being unaware of their diagnosis across world regions (Figure 2)^15–20^. People are dying because of missed opportunities to detect CKD early and deliver optimal care!

More importantly, CKD is a major risk factor for cardiovascular disease, and as kidney disease progresses, cardiovascular death and KF become competing risks^21^. Indeed, the Global Burden of Disease study data from 2019 showed that more people died of cardiovascular disease attributed to kidney dysfunction (1.7 million people) than from CKD itself (1.4 million people)^2^. Therefore, cardiovascular disease care must also be a priority for people with CKD.

## Gaps Between Knowledge and Implementation in Kidney Care

**Figure 3:**
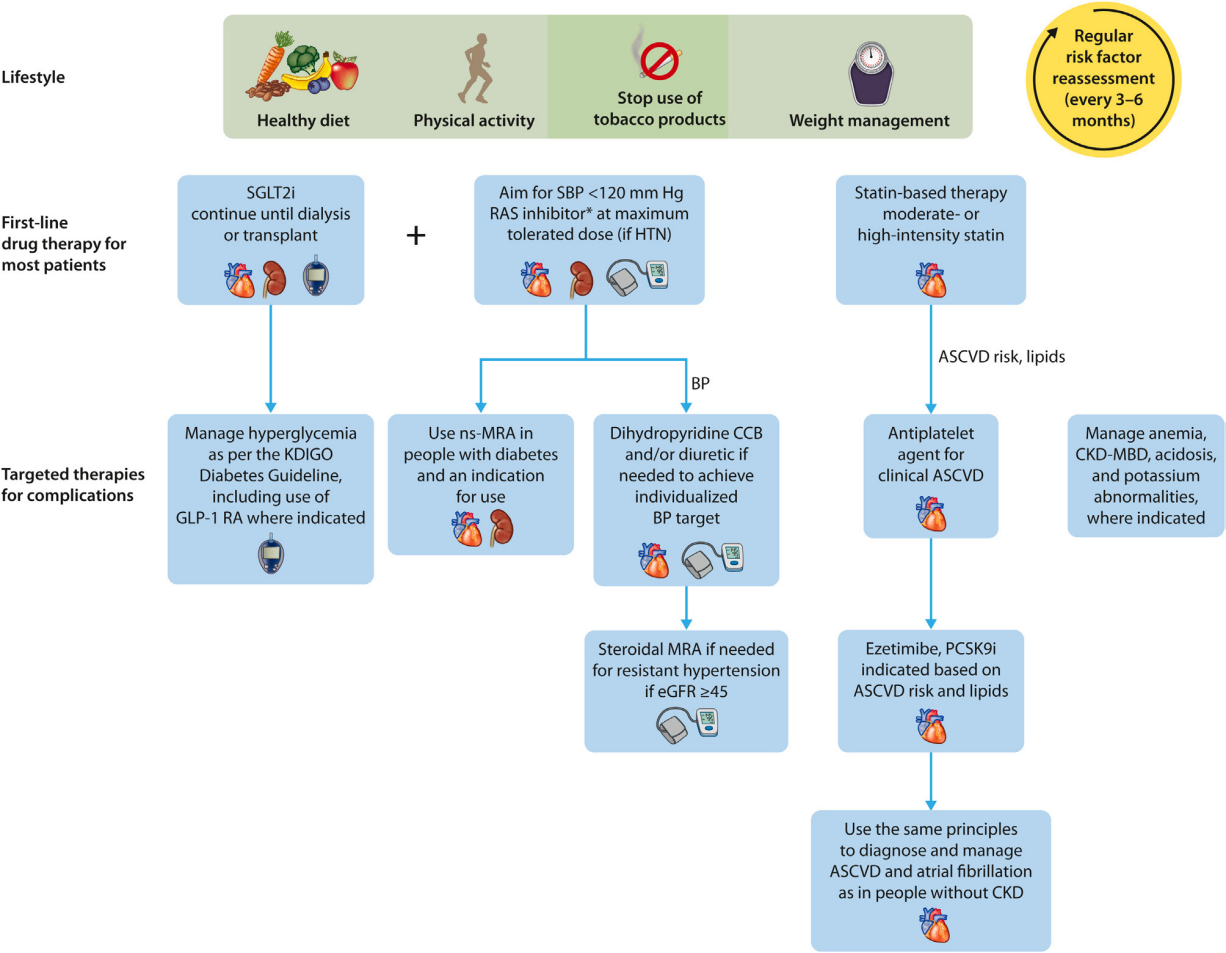
Recommended optimal lifestyle and therapeutic management for chronic kidney disease (CKD) in diabetes. Illustration of a comprehensive and holistic approach to optimal kidney health in people with CKD. In addition to the cornerstone lifestyle adjustments, attention to diabetes, blood pressure (BP), and cardiovascular risk factor control is integral to kidney care.

Strategies to prevent and treat CKD have been built on a strong evidence base over the past 3 decades (Figure 3)^19,22^. Clinical practice guidelines for CKD are clear; however, adherence to these guidelines is suboptimal (Figure 2)^15,19,20^.

Abbreviations – ASVD: atherosclerotic cardiovascular disease; CKD-MBD: chronic kidney disease-mineral and bone disorder; eGFR: estimated glomerular filtration rate; GLP-1 RA: glucagon-like peptide-1 receptor agonist; HTN: hypertension; MRA: mineralocorticoid receptor antagonist; ns-MRA: nonsteroidal mineralocorticoid receptor antagonist; PCSK9i: proprotein convertase subtilisin/ kexin type 9 inhibitor; RAS: renin-angiotensin-aldosterone system; SBP: systolic blood pressure; SGLT2i: sodium-glucose cotransporter 2 inhibitor. Notes – *Angiotensin-converting enzyme inhibitor or angiotensin II receptor blocker should be first-line theray for BP control when albuminuria is present; otherwise, dihydropyridine calcium channel blocker (CCB) or diuretic can also be considered.

Figure reproduced from Kidney Disease: Improving Global Outcomes (KDIGO) CKD Work Group. KDIGO 2024 Clinical Practice Guideline for the Evaluation and Management of Chronic Kidney Disease. *Kidney Int*. https://doi.org/10.1016/j.kint.2023.10.018 ^22^. Copyright^©^ 2023, Kidney Disease: mproving Global Outcomes (KDIGO). Published by Elsevier Inc. on behalf of the International Society of Nephrology under the CC BY-NC-ND license (https://creativecommons.org/licenses/by-nc-nd/4.0/).

Regardless of the cause, control of major risk factors, particularly diabetes and hypertension, is the foundation of optimal care for CKD^19,23^. Beyond lifestyle changes and risk factor control, the initial pharmacologic classes of agents proven to provide kidney protection were the renin-angiotensin-aldosterone system inhibitors in the form of angiotensin-converting enzyme inhibitors (ACEIs) and the angiotensin receptor blockers^14,19^. Although these medications have been known for decades to have important protective effects on kidney and heart function in people with CKD, their use has remained low based on real-world data from electronic health records (Figure 2). For example, in the United States, the use of ACEI or angiotensin receptor blocker was reported to range of 20% to 40% more than 15 years after their last approval for patients with CKD and type 2 diabetes^24^. Although more recent data show that prescribing rates have improved to 70% in this population, only 40% remain on an ACEI or angiotensin receptor blocker for at least 90 days^20^. These data illustrate gaps in both prescribing of kidney protective-medication and continuity of care over time, potentially related to cost, lack of patient education, polypharmacy, and adverse effects^25^.

Although the initial enthusiasm for sodium-glucose cotransporter 2 (SGLT2) inhibitors focused on their benefits for diabetes and cardiovascular disease, unprecedented therapeutic benefits have clearly been observed also for CKD. The relative risk reductions with SGLT2 inhibitors approach 40% for substantial decline in estimated glomerular filtration rate, KF, and death in populations with CKD of several causes, heart failure, or high cardiovascular disease risk^26,27^. These benefits accrued on top of standard-of-care risk factor management and renin-angiotensin-aldosterone system inhibitor. Risks of heart failure, cardiovascular death, and all-cause mortality were also reduced in patients with CKD^26^. Addition of SGLT2 inhibitor to renin-angiotensin-aldosterone system inhibitors could delay the need for kidney replacement therapy by several years, depending on when they are started^28^. Moreover, for every 1000 patients with CKD treated with an SGLT2 inhibitor on top of standard therapy, 83 deaths, 19 heart failure hospitalizations, 51 dialysis initiations, and 39 episodes of acute kidney function worsening can be prevented^29^.

Concerningly, there is still marked underuse of these and other guideline-recommended therapies, including SGLT2 inhibitors (Figure 2)^20,24^. In the CURE-CKD registry, only 5% and 6.3% of eligible patients with CKD and diabetes, respectively, continued on SGLT2 inhibitor and glucagon-like peptide-1 receptor agonist at 90 days^18^. Notably, lack of commercial health insurance and treatment in community-based versus academic institutions were associated with lower likelihoods of SGLT2 inhibitor, ACEI, or angiotensin receptor blocker prescriptions among patients with diabetes and CKD^20^. In low- or middle-income countries (LMICs), the gap between evidence and implementation is even wider given the high cost and inconsistent availability of these medications, despite availability of generics^30^. Such gaps in delivering optimal treatment for CKD are unacceptable.

In addition to the SGLT2 inhibitors, nonsteroidal mineralocorticoid receptor antagonists on top of the standard of care with renin-angiotensin-aldosterone system inhibitors have been demonstrated to reduce the risks of CKD progression, KF, cardiovascular events, and deaths in type 2 diabetes^31^. A growing portfolio of promising therapeutic options is on the horizon with glucagon-like peptide-1 receptor agonists (NCT03819153, NCT04865770), aldosterone synthase inhibitors (NCT05182840), and dual-to-triple incretins (Supplementary Table S1)^26,32^. Furthermore, the evidence is already clear that in patients with CKD and diabetes, glucagon-like peptide-1 receptor agonists reduce cardiovascular events, are safe and effective glucose-lowering therapies, and aid with weight loss^32^.

Historically, it has taken an average of 17 years for new treatments to move from clinical evidence to daily practice^33^. With millions of people with CKD dying each year, this is far too long a wait.

## Closing the “Gap” Between What we Know and What we do

### Lack of Policies, Global Inequities

#### Health policy

**Figure 4:**
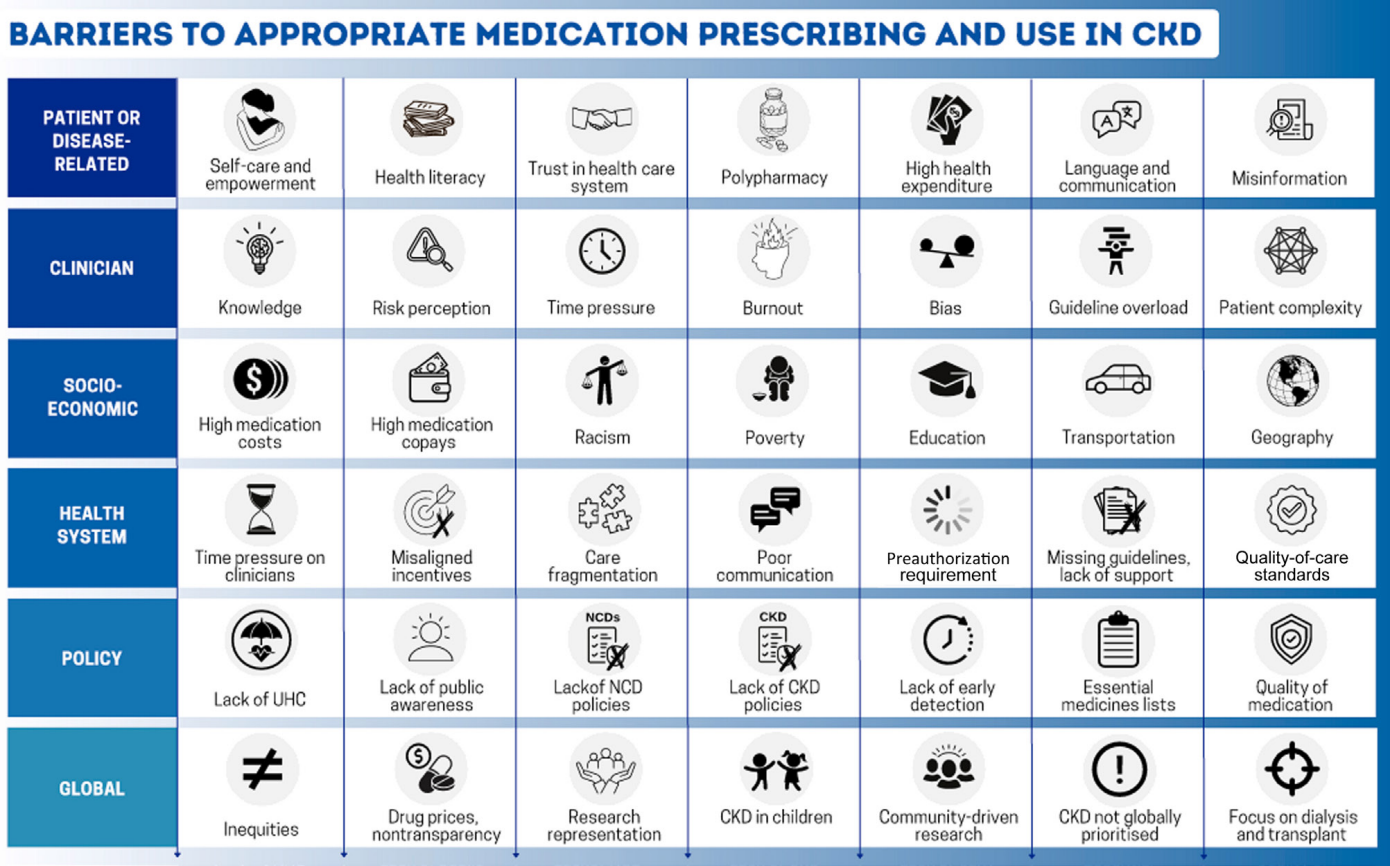
Depiction of the spectrum of factors impacting implementation of timely and quality kidney care.

Since the launch of the World Health Organization Action Plan for Non-Communicable Diseases (NCDs) in 2013, there has been global progress in the proportion of countries with a national NCD action plan and dedicated NCD units^34^. However, CKD is only incorporated into NCD strategies in approximately half of the countries^4^. Policies are required to integrate kidney care into essential health packages under universal health coverage (Figure 4)^30^. Multisectoral policies must also address the social determinants of health, which significantly increase CKD risk and severity, limiting people’s opportunities to improve their health^3^. Lack of investment in kidney health promotion, along with primary and secondary prevention of kidney disease, hinders progress^14^.

Abbreviations – CKD: chronic kidney disease; NCD: noncommunicable disease; UHC: universal health coverage.

#### Health systems

Two major goals of universal health coverage are to cover essential health services and to reduce the financial hardship imposed by health care. However, universal health coverage alone is insufficient to ensure adequate access to kidney care^3^. Health systems must be strengthened and quality of care must be prioritized, as poor quality care contributes to more deaths than lack of access in resource-poor settings^35^. Quality care requires a well-trained health care workforce, sustainable availability of accurate diagnostics, reliable infrastructure, and medication supplies and should be monitored in an ongoing process of quality improvement (Figure 4). The quality of medications, especially in LMICs, may be an additional barrier to successful management of CKD^36^. Regulation and monitoring of drug manufacturing and quality standards are important to ensure safe and effective therapies. Strategies to support regulation and quality assurance will need to be developed in the local context and guidance, as outlined elsewhere^37^.

Establishing a credible case for CKD detection and management based on risks, interventions and outcomes, and costs based on real-world data will help to translate theoretical cost-effectiveness (currently established primarily in high-income countries and with minimal data from other countries) into economic reality^30,38^. Screening should include evaluation of risk factors for CKD, taking a family history, recognizing potential symptoms (usually advanced fatigue, poor appetite, edema, itching etc.), and measuring blood pressure, serum creatinine, urinalysis, and urine albumin/protein to creatinine ratios, as outlined in established guidelines^19,39^. Addressing CKD upstream beginning in primary care should lower costs over time by reducing CKD complications and KF. Medications required for kidney care are already included in the World Health Organization Essential Medication List (Table 1). These must be provided at national level under universal health coverage^40^. Pharmaceutical companies should provide these at affordable prices.

**Table 1:** Essential medicines for patients with kidney disease

Medication/technology	Example	Reason	On WHO model list of essential medicines
ACE inhibitor	Enalapril, lisinopril	Delays CKD progression, benefits cardiovascular disease and stroke	Yes
Angiotensin receptor blocker	Losartan, telmisartan	Delays CKD progression, cardiovascular disease, and stroke	Yes
Calcium channel blocker	Amlodipine, verapamil	Blood pressure control	Yes
Loop diuretics	Furosemide, torsemide	Good when GFR is low, good for heart failure	Yes
Thiazide diuretics	Hydrochlorothiazide, metolazone, indapamide	Good for BP, especially in the Black population	Yes
SGLT2 inhibitor	Empagliflozin, canagliflozin, dapagliflozin	Diabetes control, delays CKD progression, cardiovascular disease, and death	Yes
GLP1 agonist	Semaglutide	Diabetes control, weight loss	No
Mineralocorticoid inhibitor	Spironolactone, finerenone	Delays CKD progression, reduces heart failure risk Caution: risk of hyperkalemia in patients with kidney disease	Yes/no
β-Blocker	Bisoprolol	Prevention and treatment of ischemic heart disease	Yes
Statins	Simvastatin	Prevention of CAD in patients with CKD, transplant	Yes
Aspirin		Secondary prevention of MI in patients with CKD, transplant	Yes
Fixed-dose combinations (polypill)^a^	Aspirin + atorvastatin + ramipril	Simultaneous management of CKD and cardiovascular disease and risk factors where indicated^a^	Yes
	Aspirin + simvastatin + ramipril + atenolol + hydrochlorthiazide		Yes
	Aspirin + perindopril + amlodipine		Yes
Oral hypoglycemic medication	Gliclazide, metformin, SGLT2 inhibitors	DM managementCaution with dosing and glomerular filtration rate	Yes
Insulin	Long and short acting	DM management	Yes

Abbreviations – ACE: angiotensin-converting enzyme; BP: blood pressure; CAD: coronary artery disease; CKD: chronic kidney disease; DM: diabetes mellitus; GFR: glomerular filtration rate; GLP1: glucagon-like peptide-1; MI: myocardial infarction; SGLT2: sodium-glucose cotransporter 2; WHO: World Health Organization.

Note –

a Polypills containing aspirin may not be appropriate for patients with early CKD without other cardiovascular indications.

### Challenges in Primary Care, Clinical Inertia

#### Health care professionals

The shortage of primary care professionals is exacerbated by inconsistent access to specialists and allied health professionals in both high-income countries and LMICs. Defining roles and responsibilities for kidney care is essential. Solutions may include multidisciplinary team care (primary care physicians, pharmacists, advanced practitioners, nurses, therapists, educators, nutritionists, and mental health professionals) with well-established mechanisms of collaboration between all elements and promptly available communication technologies within health systems and between professionals to support care and decision-making^41,42^. Brain drain in low-resource settings is complex and must be tackled.

The mobilization of community health workers leads to cost savings in infectious disease programs in LMICs, and may facilitate early detection, diagnosis, and management of NCDs^43^. Protocolized CKD management, possibly supported by electronic decision support systems, lends itself well to interventions at the community level, with integration of primary care physicians and backup from nephrology and other professionals^44,45^. In some environments, pharmacists could identify people with diabetes or hypertension at risk of CKD, based on their prescriptions and provide on-site testing and referral if needed^46^. Pharmacists can also provide medication reconciliation and medication advice for safety, effectiveness, and adherence. Social workers and pharmacists can help patients with medications access programs^46^.

### Challenges for Clinical Inertia

Clinical “inertia” commonly blamed for low prescription rates has many facets (Figure 4)^47^. Many knowledged gaps regarding CKD exist among primary care clinicians^48^. Such gaps can be closed with targeted public and professional education. Additional factors include fear of adverse drug effects, misaligned incentives within the health system, excessive workload, formulary restrictions, and clinician burnout^47^. Furthermore, discrepancies in guideline recommendations from different professional organizations may add to the confusion. A major impediment to optimal care is the time constraints imposed on individual clinicians. The average primary care practitioner in the United States would require ≈26.7 hours per day to implement guideline-recommended care for a 2500 patient panel^49^. Innovation is required to support guideline implementation, especially for primary care practitioners who must implement many different guidelines to meet the needs of various patients. Electronic health records, reminders, team-based nudges, and decision support tools offer a promising support for quality kidney care in busy clinical practices^50^. The extra time and effort spent negotiating preauthorizations or completing medication assistance program requests, along with need for frequent monitoring of multiple medications also hinder appropriate prescribing^25^. Many primary care practitioners have only a few minutes allocated per patient because of institutional pressure or patient volume. “Inertia” can hardly be applied to clinicians working at this pace. The number of health professionals must increase globally.

Visits for patients with CKD are complex as multimorbidity is high. Patients are often managed by multiple specialists, leading to fragmentation of care, lack of holistic oversight, and diffusion of responsibility for treatment. Multidisciplinary care improved transition to kidney replacement therapy and lowered mortality in single and combined outcome analyses^51^. Novel models of “combined clinics” with on-site collaboration and coparticipation (nephrologist-cardiologist-endocrinologist) may prove highly beneficial for patients by reducing fragmentation of care, improving logistics, and saving costs.

### Patient Centeredness

#### Health literacy

Self-care is the most important aspect of kidney care. A patient’s ability to understand his/her health needs, make healthy choices, and feel safe and respected in the health system, and psychosocial support are important to promote health decision-making (Figure 4). Good communication requires quality information and, above all, confirmation of “understanding” on the part of the patient and often the family. Electronic apps and reminders can be useful tools to support patients by improving disease knowledge, promoting patient empowerment, and improving self-efficacy, although it is unlikely that “one size will fit all”^52^. Insufficient patient health information, poor communication, and mistrust, among other elements, are important barriers, especially in marginalized and minoritized communities, where CKD is common^30^. Patients may also be confused by contradictory care recommendations between healthcare professionals, as well as conflicting messaging in lay media. Innovative platforms to improve communication between patients and clinicians about CKD are promising and may promote optimal prescribing and adherence^53,54^.

To overcome barriers and promote equity, patient perspectives are essential to designing and testing better health strategies. Collaborative care models must include patients, families, community groups, diverse health care professionals, health systems, government agencies, and payers^38^. Advocacy organizations and local community groups and peer navigators, having trusted voices and relationships, can help educate and provide input for development of patient tools and outreach programs^55^. Most importantly, patients must be at the center of their care.

#### Cost and availability of medication

In high-income countries, people without health insurance and those with high copays paradoxically pay the most for even essential medications^38^. Across LMICs, kidney disease is the leading cause of catastrophic health expenditure because of reliance on out-of-pocket payments^56^. Across 18 countries, 4 cardiovascular disease medications (statins, ACEIs, aspirin, and *β*-blockers), all often indicated in CKD, were more available in private than in public settings, mostly unavailable in rural communities, and unaffordable for 25% of people in upper middle-income countries and 60% of people in low-income countries^57^. Newer therapies may be prohibitively expensive worldwide, especially where generics may not yet be available. In the United States, the retail price for a 1-month supply of an SGLT2 inhibitor or finerenone is ≈US$ 500 to US$ 700; and for glucagon-like peptide-1 receptor agonists, ≈US$ 800 to US$ 1200 per month^38^. Reliance on out-of-pocket payment for vital, life-saving basic medications is unacceptable (Figure 4).

### Special Considerations

Not all kidney diseases are the same. Much of what has been discussed here relates to the most common forms of CKD (e.g. diabetes and hypertension). Some forms of CKD not yet completely understood have different risk profiles, including environmental exposures, genetic predisposition, and autoimmune or other systemic disorders. Highly specialized therapies may be required. Pharmaceutical companies should be responsible for ensuring that research studies include disease-representative participants with appropriate race, ethnicity, and sex and gender representation, that effective drugs are made available after studies, and that the balance between profits and prices is fair and transparent. Many novel therapies offer new hope for diverse kidney diseases; and once approved, there must be no delay in extending the benefits to all affected patients (Supplementary Table S1).

An important group often overlooked is children with kidney diseases. This group is especially vulnerable in LMICs, where nephrology services and resources are limited, and families must often make the choice of paying for treatment for one child or supporting the rest of the family^58^. Children with CKD are also at high risk of cardiovascular disease, even in high-income settings, and more attention is required to control risk factors and achieve treatment targets^59^.

### Fostering Innovation

#### Implementation science and knowledge translation

Given that we know how to treat CKD based on a rigorous evidence base, we must now optimize implementation^60^. Implementation research aims to identify effective solutions by understanding how evidence-based practices, often developed in high-income countries, can be integrated into care pathways in lower-resource settings. The management of CKD lends itself to implementation research: optimal therapeutic strategies are known, outcomes are easily measurable, and essential diagnostics and medications should already be in place. Eliciting local patient preferences and understanding challenges are crucial components of such research. Ministries of health should commit to overcoming identified barriers and scaling up successful and sustainable programs.

#### Polypills as an example of simple innovation

Polypills are attractive on multiple levels: fixed doses of several guideline-recommended medications are present within one tablet (Table 1), the price is lower, the pill burden is lower, and the regimen is simple^61^. Polypills have been shown to prevent cardiovascular disease and are cost-effective for patients with CKD^62^. More studies are needed, but given the alternatives of costly kidney replacement therapy or early death, it is likely that polypills will prove cost-effective in reducing CKD progression.

#### Harnessing digital technologies

Integration of telehealth and other types of remotely delivered care can improve efficiency and reduce costs^63^. Electronic health records and registries can support monitoring quality of care, identify gaps to guide implementation, and improve outcomes within learning healthcare systems. Artificial intelligence can also be used for risk stratification and personalization of medication prescribing and adherence^64^. The use of telenephrology for communication between primary care and subspecialists may also prove useful and beneficial for patient treatment^65^.

### Patient Perspectives

There are multiple methods for eliciting patient preferences for CKD care, including interviews, focus groups, surveys, discrete choice experiments, structured tools, and simple conversations^66,67^. At present, many of these methods are in research stages. Translation into the clinic will require contextualization and determination of local and individual acceptability.

The journey of each person living with CKD is unique, but it is combined with challenges and barriers. As examples of lived experiences, comments from patients about their medications and care are outlined in Chart 1 and Supplementary Table S2. These voices must be heard and followed to close gaps and improve quality of kidney care everywhere.

**Chart 1:** Barriers impacting medication use as expressed by people living with kidney disease

“I have to pay for my medications so I either settle for less expensive options or ration the regular dose.” “I am seeing doctors of different specialties each of whom prescribe separate regimens which makes me concerned about drug interactions.” “As an experienced patient, I sometimes stop, or modify the dose of the prescribed medications without referring to my doctors. If they do ask, I would tell them that I am in full compliance.” “Over time, the dose and varieties of my medication keep increasing. I am not sure whether it’s because of condition worsening or medications becoming less effectiveness.” “My knowledge of medication mostly comes from a peer patient who appears to be very knowledgeable about this stuff.”

#### Call to Action

A stalemate in kidney care has been tolerated for far too long. The new therapeutic advances offer real hope that many people with CKD can survive without developing KF. The evidence of clinical benefit is overwhelming and unequivocal. We cannot wait another 17 years for this evidence to be translated into clinical practice^33^. Now is the time to ensure that everyone who is eligible to receive CKD treatment equitably receives it.

Known barriers and global disparities in access to diagnosis and treatment must be urgently addressed (Figure 4). To achieve health equity for people with kidney diseases and at-risk patients, we must raise awareness from policy makers to patients and the general population, harness innovative strategies to support all cadres of healthcare workers, and balance profits with reasonable prices (Table 2). If we narrow the gap between what we know and what we do, kidney health will become a reality worldwide.

**Table 2:** Examples of strategies to improve implementation of appropriate CKD care

Domain	Potential solutions
Health policy	Include NCD and CKD as health care priorities; ensure sustainable financing; monitor disease burdens and outcomes; registries; multisectoral action; promote kidney health through public health measures; achieve SDGs
Health systems	Integrate CKD care into primary care under UHC; establish quality standards; include necessary diagnostics and medications in national essential medication/diagnostic lists; monitoring and evaluation; reduce brain drain; monitor equity; simplify and streamline guidelines
Quality assurance	Regulation and monitoring of medication quality, especially of generics. Monitoring of health outcomes and care processes to permit iterative improvement
Health care professionals	Reduce time pressure; improve knowledge; broaden scope of practice (e.g. pharmacists); engage community health workers
Patient empowerment	Health literacy; education; community engagement; involvement in research design and conduct
Medication cost	Quality generics; reduce prices; UHC for essential medications
Implementation research	Identify barriers within local contexts; test solutions to overcome barriers
Polypills	Reduce cost; lower pill burden
Digital technologies	Electronic pill boxes, bags, bottles; blister pack technology; ingestible sensors; electronic medication management systems; patient self-report technology; video-based technology; motion sensor technology; telemedicine; smartphone apps; electronic health records

Abbreviations – CKD: chronic kidney disease; NCD: noncommunicable disease; SDG: sustainable development goal; UHC: universal health coverage.

## Disclosure

VL is chair of the Advocacy Working Group, International Society of Nephrology, no financial disclosures. KRT has received research grants from the National Institutes of Health (National Institute of Diabetes and Digestive and Kidney Diseases, National Heart, Lung, and Blood Institute, National Center for Advancing Translational Sciences, National Institute on Minority Health and Health Disparities, director’s office), the US Centers for Disease Control and Prevention, and Travere Therapeutics, and consultancy fees from AstraZeneca, Bayer, Boehringer Ingelheim, Eli Lilly, and Novo Nordisk. She is the chair of the Diabetic Kidney Disease Collaborative for the American Society of Nephrology. RC-R is a member of the Steering Committee of World Kidney Day, a member of the Diabetes Committee of the Latin-American Society of Nephrology and Hypertension (SLANH), and a member of the Latin American Regional Board, International Society of Nephrology. He is a member of the Steering Committee of the Dapagliflozin and Prevention of Adverse Outcomes in Chronic Kidney Disease (DAPA-CKD) trial (AstraZeneca), the Study of Diabetic Nephropathy with Atrasentan (SONAR) (Abbvie), A Non-interventional Study Providing Insights Into the Use of Finerenone in a Routine Clinical Setting (FINE-REAL) (Bayer), and CKD-ASI (Boehringer). He has received research grants from AstraZeneca, GlaxoSmithKline, Roche, Boehringer, and Novo Nordisk; and has received honoraria as a speaker from AstraZeneca, Bayer, Boehringer Ingelheim, and Amgen. All the other authors declared no competing interests.

## Supplementary Material

The following online material is available for this article:

Figure S1 - Ranking of kidney dysfunction as a cause of death stratified by world-income category and gender by level 2 risk factors for death.

Table S1 - Approved and emerging novel therapeutic agents for various kidney diseases.

Table S2 - Patient comments on accessibility, affordability, knowledge, facilitators and barriers to optimal kidney care.
