# Supplementary material for: Mind the gap in kidney care: translating what we know into what we do
Source: J Bras Nefrol. 2024 Jul 5;46(3):e2024E007. doi: 10.1590/2175-8239-JBN-2024-E007en (PMC11239182; doi:10.1590/2175-8239-JBN-2024-E007en)
Supplement: Supplementary file 1 [file 2175-8239-jbn-46-3-e2024E007-suppl1.pdf]

Braz. J. Nephrol. (J. Bras. Nefrol.) 2024, 46(3):e2024E007
https://doi.org/10.1590/2175-8239-JBN-2024-E007en

Supplementary Material to "Mind the gap in kidney care: translating what we know into what we do"

Figure S1. Ranking of kidney dysfunction as a cause of death stratified by world-income category and gender by level 2 risk factors for death.

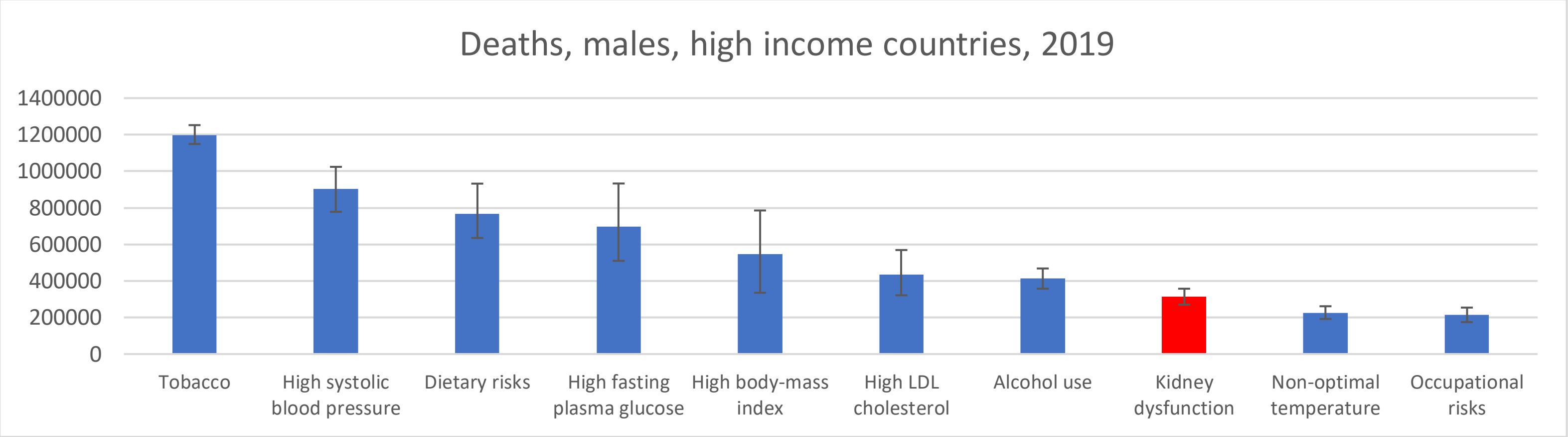

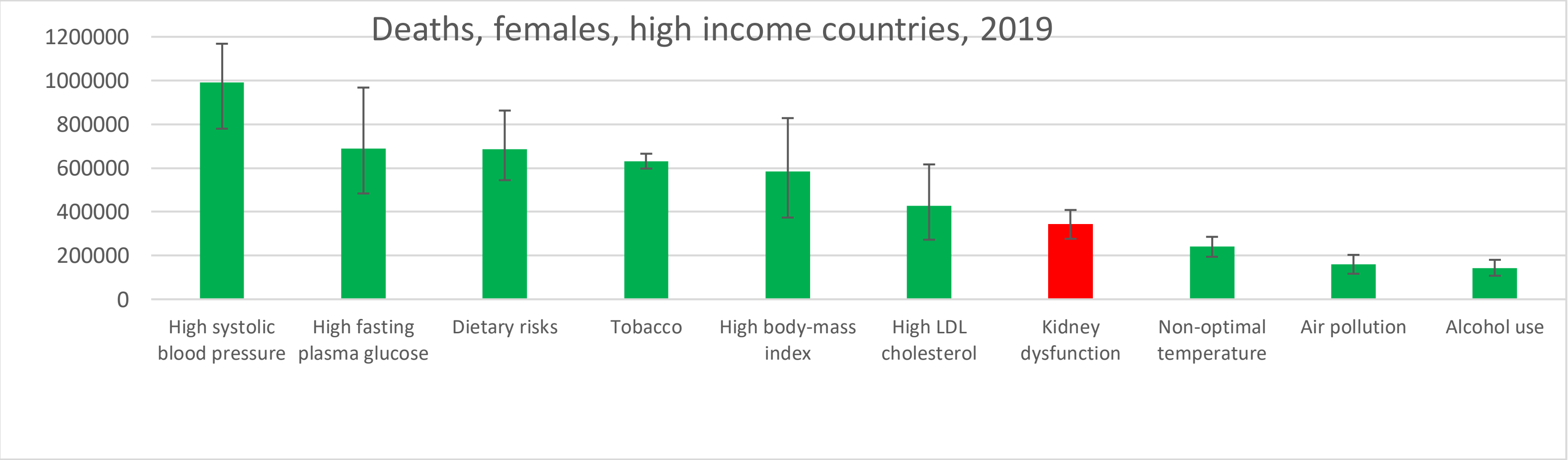

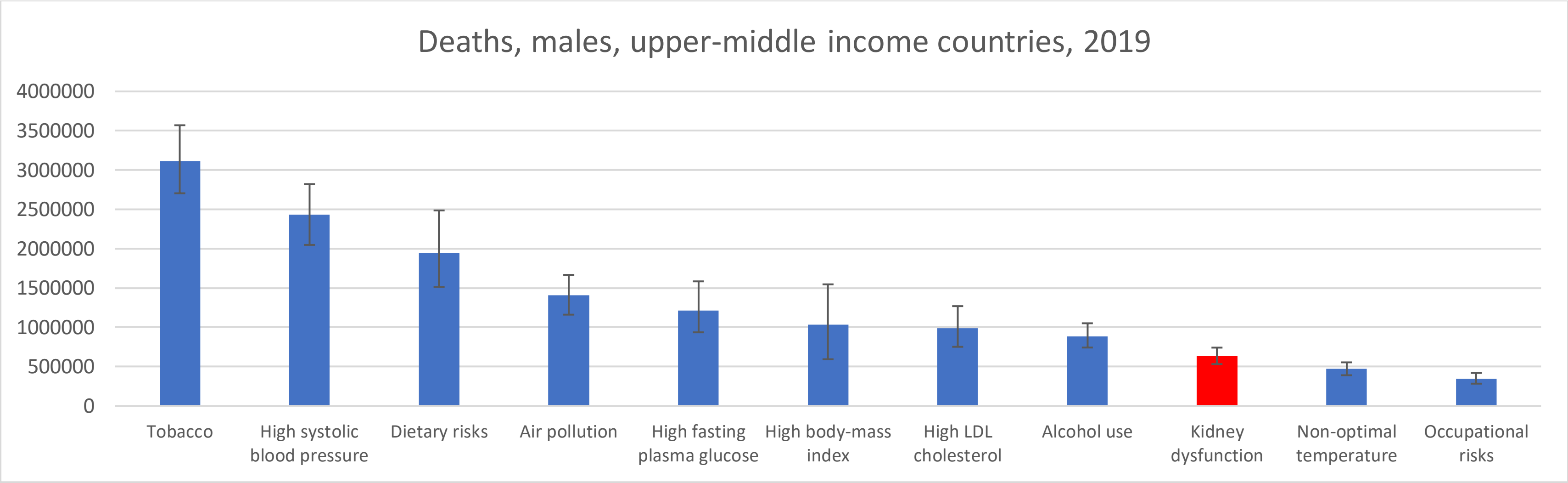
Deaths, males, upper-middle income countries, 2019
4000000
3500000
3000000
2500000
2000000
1500000
1000000
500000
0
Tobacco
High systolic blood pressure
Dietary risks
Air pollution
High fasting plasma glucose
High body-mass index
High LDL cholesterol
Alcohol use
Kidney dysfunction
Non-optimal temperature
Occupational risks

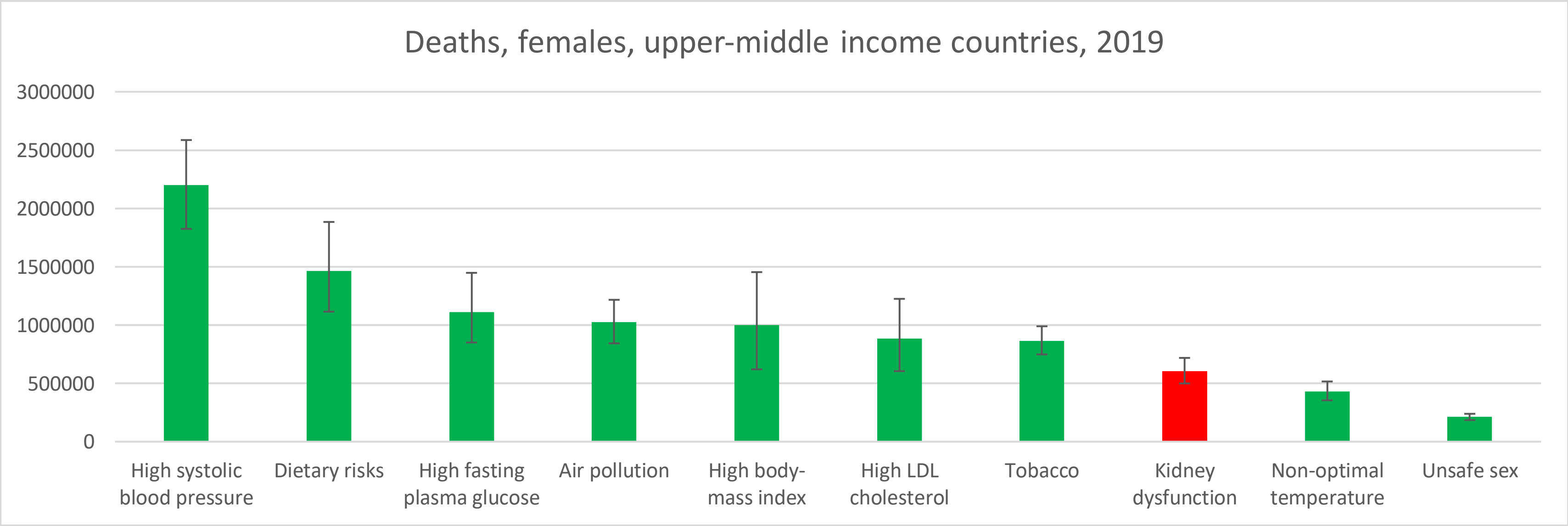
Deaths, females, upper-middle income countries, 2019
3000000
2500000
2000000
1500000
1000000
500000
0
High systolic blood pressure
Dietary risks
High fasting plasma glucose
Air pollution
High body-mass index
High LDL cholesterol
Tobacco
Kidney dysfunction
Non-optimal temperature
Unsafe sex

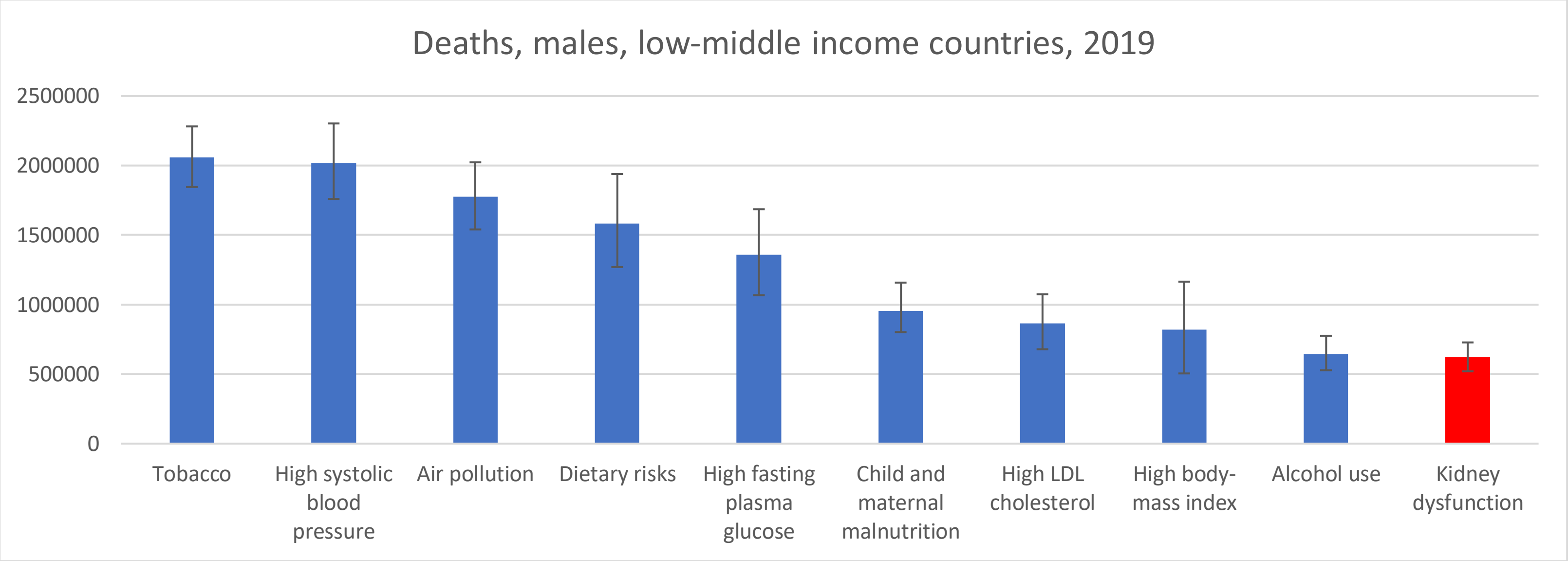
Deaths, males, low-middle income countries, 2019
2500000
2000000
1500000
1000000
500000
0
Tobacco
High systolic blood pressure
Air pollution
Dietary risks
High fasting plasma glucose
Child and maternal malnutrition
High LDL cholesterol
High body-mass index
Alcohol use
Kidney dysfunction

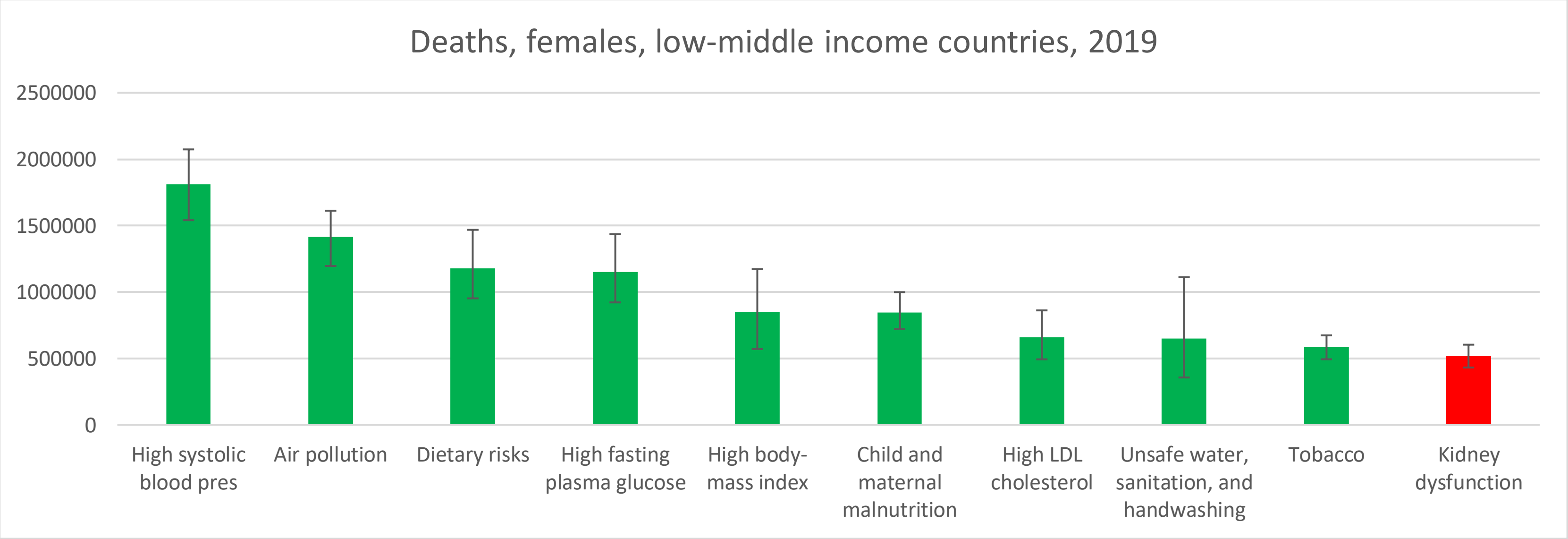
Deaths, females, low-middle income countries, 2019
2500000
2000000
1500000
1000000
500000
0
High systolic blood pres
Air pollution
Dietary risks
High fasting plasma glucose
High body-mass index
Child and maternal malnutrition
High LDL cholesterol
Unsafe water, sanitation, and handwashing
Tobacco
Kidney dysfunction

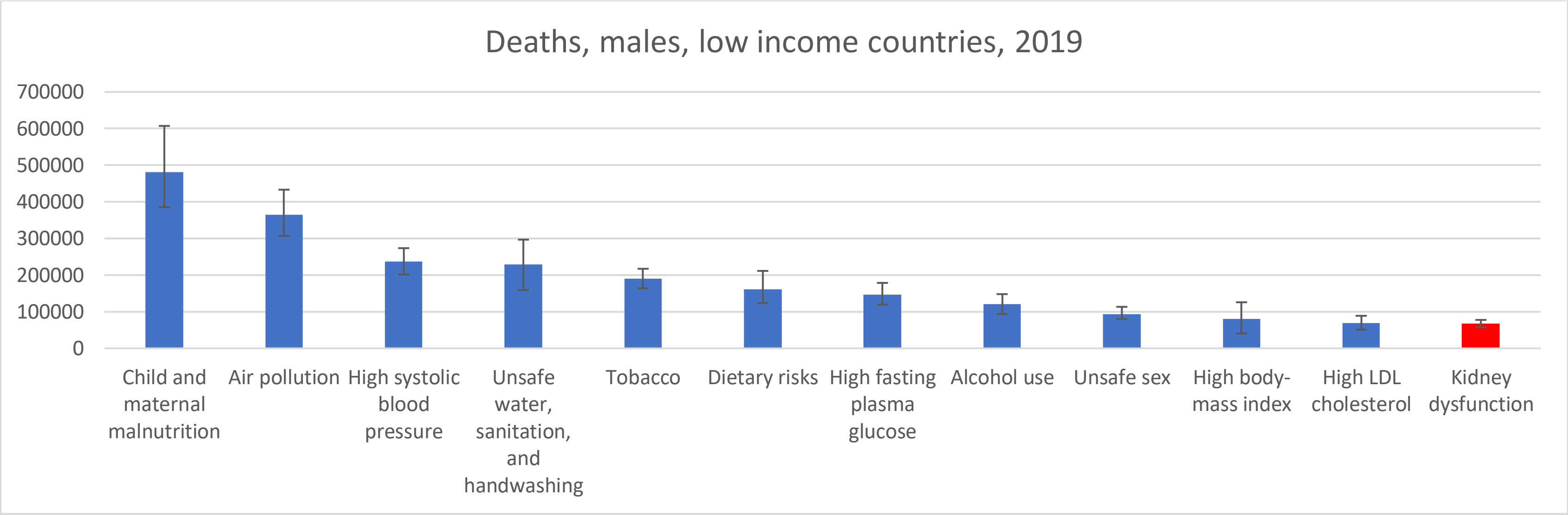

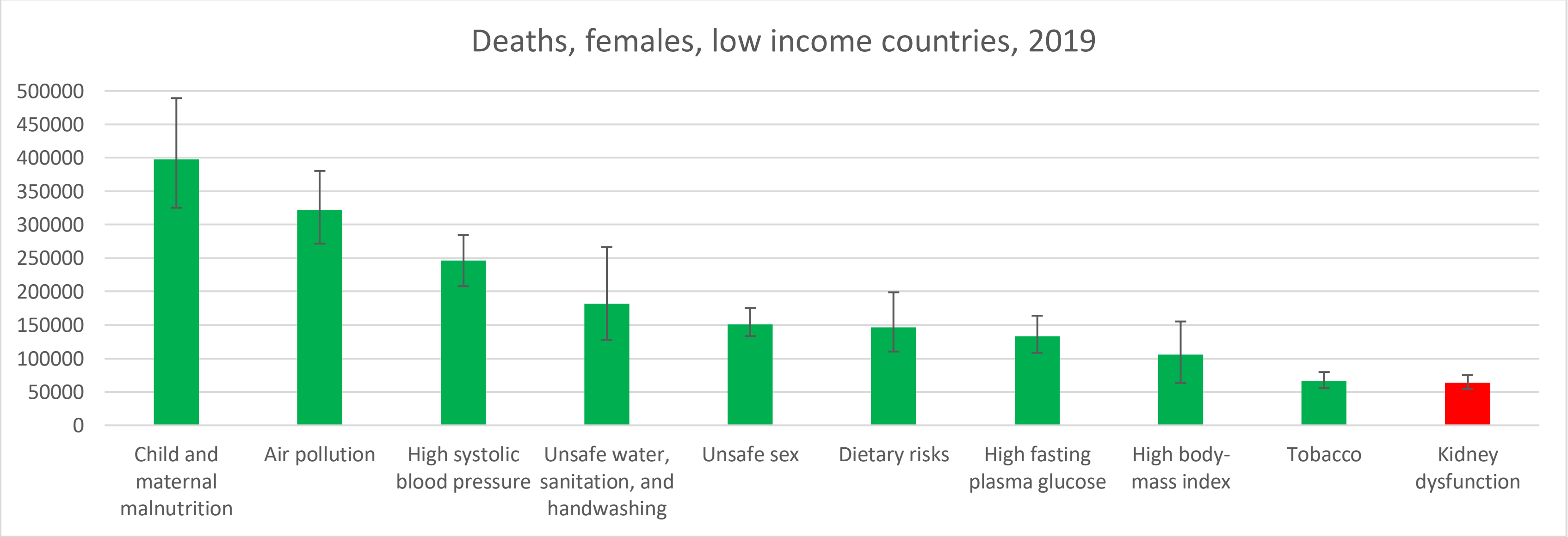

Data form Global Burden of Diseases Study: https://vizhub.healthdata.org/gbd-results/
