# Supplementary material for: Mind the gap in kidney care: translating what we know into what we do
Source: J Bras Nefrol. 2024 Jul 5;46(3):e2024E007. doi: 10.1590/2175-8239-JBN-2024-E007en (PMC11239182; doi:10.1590/2175-8239-JBN-2024-E007en)
Supplement: Supplementary file 2 [file 2175-8239-jbn-46-3-e2024E007-suppl2.pdf]

Braz. J. Nephrol. (J. Bras. Nefrol.) 2024, 46(3):e2024E007
https://doi.org/10.1590/2175-8239-JBN-2024-E007en

Supplementary Material to "Mind the gap in kidney care: translating what we know into what we do"

TABLE S1 Approved and emerging novel therapeutic agents for various kidney diseases.

Medications (Class)	Conditions	Status	Clinical Trials
Inaxaplin (APOL1 inhibitor)	Two APOL1 variants FSGS	Emerging	NCT04340362
Sparsentan (Endothelin A receptor antagonist)	FSGS	Emerging	DUPLEX (NCT03493685)
	IgAN	Approved	PROTECT (NCT03762850)
Budesonide (Corticosteroid)	IgAN	Approved	NEFIGARD (NCT03643965)
Atrasentan (Endothelin A receptor antagonist)	IgAN	Emerging	ALIGN (NCT04573478)
Sibeprenlimab (APRIL inhibitor)	IgAN	Emerging	VISIONARY (NCT05248646)
Narsoplimab (MASP-2 inhibitor)	IgAN	Emerging	ARTEMIS-IgAN (NCT02682407)
Iptacopan (Factor B inhibitor)	IgAN	Emerging	APPLAUSE-IgAN (NCT04578834)
	aHUS	Emerging	APPELHUS (NCT04889430)
Ravulizumab (long acting anti-C5 monoclonal antibody)	aHUS	Approved	ALXN1210-aHUS311 (NCT02949128)
Dapagliflozin/ Empagliflozin (SGLT2-i)	CKD/ DKD	Approved	DAPA-CKD (NCT03036150)<br>EMPA-kidney (NCT03594110)
Canagliflozin (SGLT2-i)	DKD	Approved	CREDENCE (NCT02065791)
Finerenone (non-steroidal mineralocorticoid receptor antagonists)	DKD	Approved	FIGARO-DKD (NCT02545049)<br>FIDELIO-DKD (NCT02540993)
	CKD	Emerging	FIND-CKD (NCT05047263)
Ziltivekimab (Antibody Mediated IL-6 Inhibition)	CKD	Emerging	RESCUE (NCT03926117)<br>RESCUE II (NCT04626505)
	Atherosclerotic CVD in CKD		ZEUS (NCT05021835)
Semaglutide (GLP-1 receptor agonist)	DKD	Emerging	FLOW (NCT03819153)

**Medications (Class)**	**Conditions**	**Status**	**Clinical Trials**
Voclosporin (Calcineurin inhibitor)	Lupus nephritis	Approved	AURORA1/2 (NCT03021499)
Belimumab (anti-BAFF monoclonal antibody)	Lupus nephritis	Approved	BLISS-LN (NCT01639339)
Anifrolumab (anti-interferon α antibody)	Lupus nephritis	Emerging	IRIS (NCT05138133)
Daratumumab (anti-CD38 monoclonal antibody)	Lupus nephritis	Emerging	NCT04868838
Avacopan (C5a receptor antagonist)	ANCA vasculitis	Approved	ADVOCATE (NCT02994927)

aHUS, atypical haemolytic uremic syndrome; CKD, chronic kidney disease; DKD, diabetic kidney disease; FSGS, focal segmental glomerulosclerosis; IgAN, IgA nephropathy
