# Supplementary material for: Mind the gap in kidney care: translating what we know into what we do
Source: J Bras Nefrol. 2024 Jul 5;46(3):e2024E007. doi: 10.1590/2175-8239-JBN-2024-E007en (PMC11239182; doi:10.1590/2175-8239-JBN-2024-E007en)
Supplement: Supplementary file 3 [file 2175-8239-jbn-46-3-e2024E007-suppl3.pdf]

Braz. J. Nephrol. (J. Bras. Nefrol.) 2024, 46(3):e2024E007
https://doi.org/10.1590/2175-8239-JBN-2024-E007en

Supplementary Material to "Mind the gap in kidney care: translating what we know into what we do"

TABLE S2 Patient comments on accessibility, affordability, knowledge, facilitators and barriers to optimal kidney care.

**Patient comment/category**		**Cost**	**Side effects, interaction**	**Patient empowermen**	**Safety**	**Source of information**	**Comfort level to query**	**Delay new therapy**	**New hope**	**Policies/ prevention**	**Education**	**Fragmentati on of care**	**Lack of specialists**
**HK 1**	The medication is not covered by UHC or insurance policies, I cannot afford to take the most effective medications as recommended by my doctors. I decide to not to take the medication, settle for less expensive option or start to ration the regular dose.	x											
**HK 2**	Given the side effects, not taking medication to the extent possible would be the best policy.		x										
**HK 3**	I do not feel any side effects and question about the medication effectiveness.		x	x									
**HK 4**	I am concerned or unsure about the interactions among the medications taken especially asI am seeing doctors of different specialties each of whom prescribe separate regimens of their own.		x										
**HK 5**	I can stop taking my medication when my laboratory results improve or when I start to feel better.			x									
**HK 6**	All over-the-counter medications are generally safe for me to take.		x								x		
**HK 7**	The dose and varieties of medication keep increasing. I am not sure whether it's because of condition worsening or less effectiveness of medication.		x										
**HK 8**	I am sceptical about adding new medications to my existing regimen and have the natural tendency of resisting any new additions or dose increase.				x								
**HK 9**	As an experienced patient, I sometimes stop, or adjust the dose of the medication prescribed without telling my doctors. Or if they do ask, I would tell them that I am in full compliance.			x									
**HK 10**	My knowledge of medication mostly comes from a peer patient who appears to be very knowledgeable about this stuff.					x							
**UK 1**	People need confidence to tell clinicians about side effects of medications and clinicians can offer education and encouragement to explain medications (and also encourage people not to stop taking them without discussion)		x				x						
**UK 2**	In England we have prescription charges on certain medications unless you are on dialysis. These charges, about £10 each and can be a barrier for some. Yet in Scotland, Wales, and Northern Ireland there are no charges.	x											
**UK 3**	New medicines can take a very long time to reach patients even after approval from the regulators as payers can be reluctant							x					

Patient comment/category		Cost	Side effects, interaction	Patient empowermen	Safety	Source of information	Comfort level to query	Delay new therapy	New hope	Policies/ prevention	Education	Fragmentati on of care	Lack of specialists
**UK 4**	New medications such as those which may delay CKD have been greeted with excitement by many.								x				
**UK 5**	A universal focus on preventative approaches to CKD, including exercise, diet, emotional support as well as medications is one that's likely to be most patient-centred but needs a plan.									x			
**HN 1**	From the perspective of kidney patients in Honduras/Latin America, access to medicines is crucial to our quality of life and survival. For this we mention some points of importance related to access to medicines and what this means, based on our current experience based on the theme of World Kidney Day 2024, "Kidney health for all: Advancing equitable access to care and optimal medication practice "	x											
**HN**	In the absence of solid programs/policies regarding the management of comprehensive kidney disease, as in Honduras, isolated programs exist for renal care, where the action is mostly geared to dialysis treatment only. Government responsibility for public assistance covers 85% of the renal population, 12-13% is covered by the social security system, and about 3% privately. Currently in Honduras, the law that does not cover the interests of kidney patients and it must be updated and revised in order to manage the comprehensive program of kidney disease.	x								x			
**HN**	There is a significant diversity observed in the other countries of Latin America in health policies regarding Renal Health Programs promoted by SLANH, however many of them have shortcomings ranging from timely and early diagnosis, lack of coverage and accessibility to medicines. In Latin America, in recent years n most countries the focusing on the accessibility of medication has been on price rather than the benefit, effectiveness and quality of medicines.	x								x			
**HN**	In Honduras, access to medicines is limited due to economic barriers and the type of health system that is managed. Many patients struggle to obtain the medications they need, but they cannot always be obtained and the costs of the low-income patient are inaccessible. Even though the Ministry of Health has a basic set of medicines, it does not meet the needs and does not adjust to the growth of the renal population, therefore they are insufficient.	x											
**HN**	In Honduras, the absence of programs defined in primary care for the early or opportune detection of chronic diseases such as kidney, and having a fairly collapsed health system, has led to the increases in kidney disease in the last decade, and this has been deepened with the lack of health budget. ItFor these reasons patients are unable to obtain appropriate treatments of quality and with the regulatory requirements that are required. On the other hand, the lack of specialized professional resources affects us directly since medical cares is given only in case of extreme urgency and this means that in many cases very advanced complications are already detected.	x								x			

Patient comment/category		Cost	Side effects, interaction	Patient empowermen	Safety	Source of information	Comfort level to query	Delay new therapy	New hope	Policies/ prevention	Education	Fragmentati on of care	Lack of specialists
**HN**	In Latin America, some countries which have developed a national public health coverage such as Argentina and Uruguay do not have major deficits but the rest of the countries have many deficiencies within their public systems in terms of timely medical attention. There is lack of education regarding the disease for patients.	x								x	x		
**HN**	We have very few specialized health professionals in the renal area and most are concentrated in the main cities of the country in Honduras, little or none in the other areas where the number of patients affected by kidney disease is growing, and this directly affects the patient since they have to migrate to the cities to be able to be treated affecting their quality of life or they have to abandon treatment for not having the resources. Although in Honduras in recent years we have had an increase in renal specialists, these are concentrated in two areas of the country leaving uncovered the other 16 areas. Policies are needed to help improve this shortage, to improve care, especially in the hospitals of the network of the Secretary of Health. Care accessibility barriers need to be broken.									x			x
**HN**	In Honduras in recent years we have managed to organize some groups or NGOs of patients who fight for improvements in accessibility to adequate and timely medication with the quality of the same under the necessary regulatory standards, but this is only small percentages of the affected population, that is why we fight in education for patient empowerment, However, we often find barriers and paradigms on the part of medical societies and health authorities, but we have achieved and made written approaches, as well as little by little the involvement with entities that have to do directly with the provision of services and management of medicines.			x									
**HN**	At the Latin American level, we work on empowerment on the different actions necessary to address renal health programs through scientific societies in different educational aspects from the perspective of the patient and health professionals, trying to make joint work alliances to raise awareness about the underlying diseases that may lead to kidney disease. All this work through participating in networks of associations at national and international level.			x							x		
**HN**	In summary, access to medicines is essential for kidney patients in Honduras, as these medicines not only relieve pain and discomfort, but are also critical to preventing serious complications and maintaining an acceptable quality of life. Ensuring equitable access to care and medication is essential to addressing the kidney health needs of all patients in the country. And at the same time through the different actions and struggles that are executed, empower us to be able to participate in the different health policies necessary to have dignified and adequate medical care with accessibility to the best health practices by renal specialists and the appropriate effective and quality timely medication.	x											
**IN1**	Patients are well advised on need for medications at all stages. When prescription changes more information on need for change for better outcome is needed.										x		

Patient comment/category		Cost	Side effects, interaction	Patient empowermen	Safety	Source of information	Comfort level to query	Delay new therapy	New hope	Policies/ prevention	Education	Fragmentati on of care	Lack of specialists
**IN2**	Co-relation between medications and the dietary requirements need to be established		x		x								
**IN3**	Cost is a huge factor for the poor as well as middle -income patients	x											
**IN4**	Exceptional drugs for rare diseases like eculizumab must be made available at reasonable costs world-wide	x								x			
