# Supplementary material for: Mind the gap in kidney care: translating what we know into what we do
Source: J Bras Nefrol. 2024 Jul 5;46(3):e2024E007. doi: 10.1590/2175-8239-JBN-2024-E007en (PMC11239182; doi:10.1590/2175-8239-JBN-2024-E007en)
Supplement: Supplementary file 4 [file 2175-8239-jbn-46-3-e2024E007-suppl4.pdf]

Braz. J. Nephrol. (J. Bras. Nefrol.) 2024, 46(3): e2024E007
https://doi.org/10.1590/2175-8239-JBN-2024-E007pt

Material Suplementar para “Atenção às lacunas no cuidado renal: traduzindo o que sabemos em ações”

Figura S1. Classificação da disfunção renal como causa de óbito estratificada por categoria de renda mundial e sexo, por fatores de risco de nível 2 para óbito.

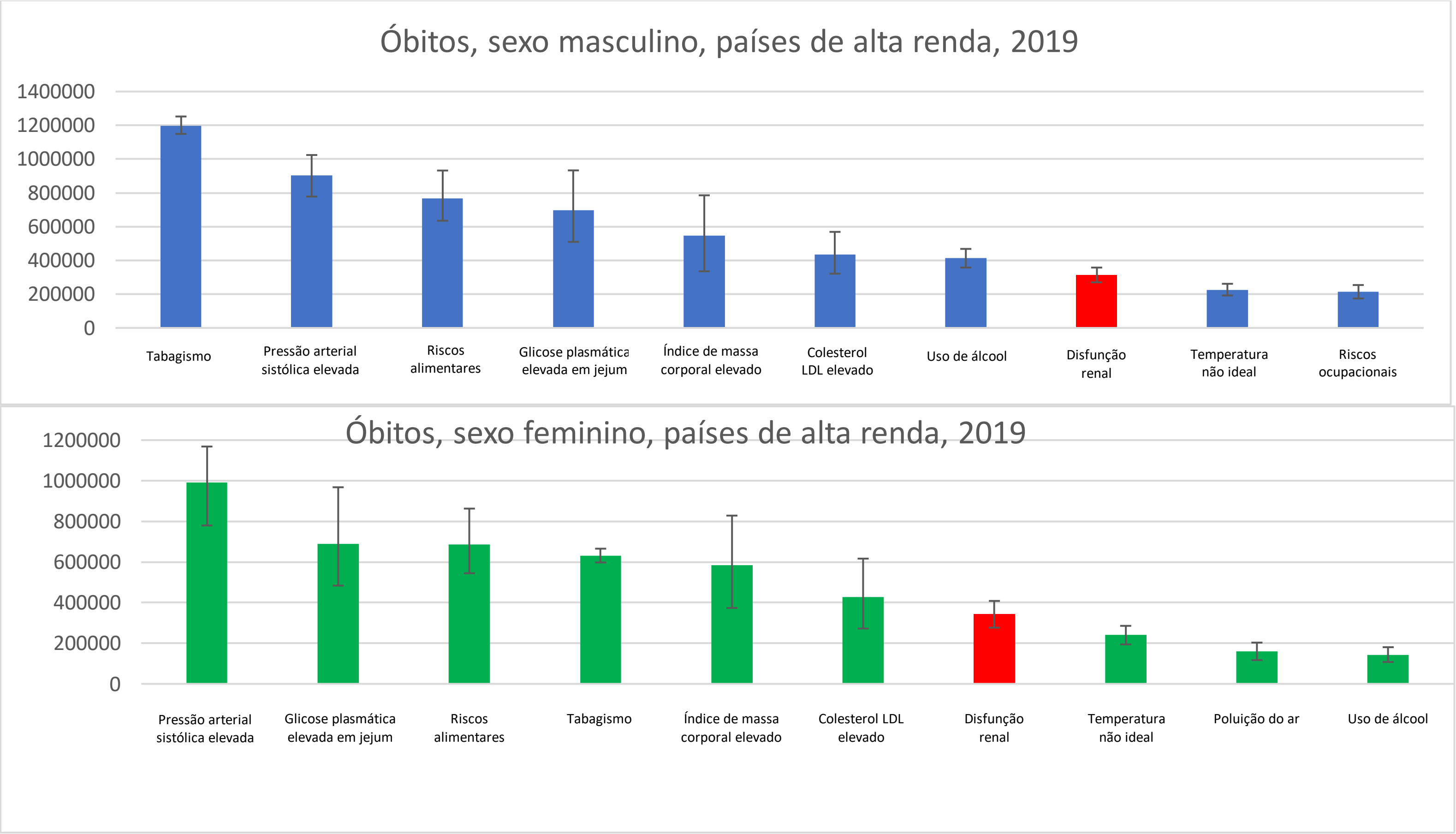

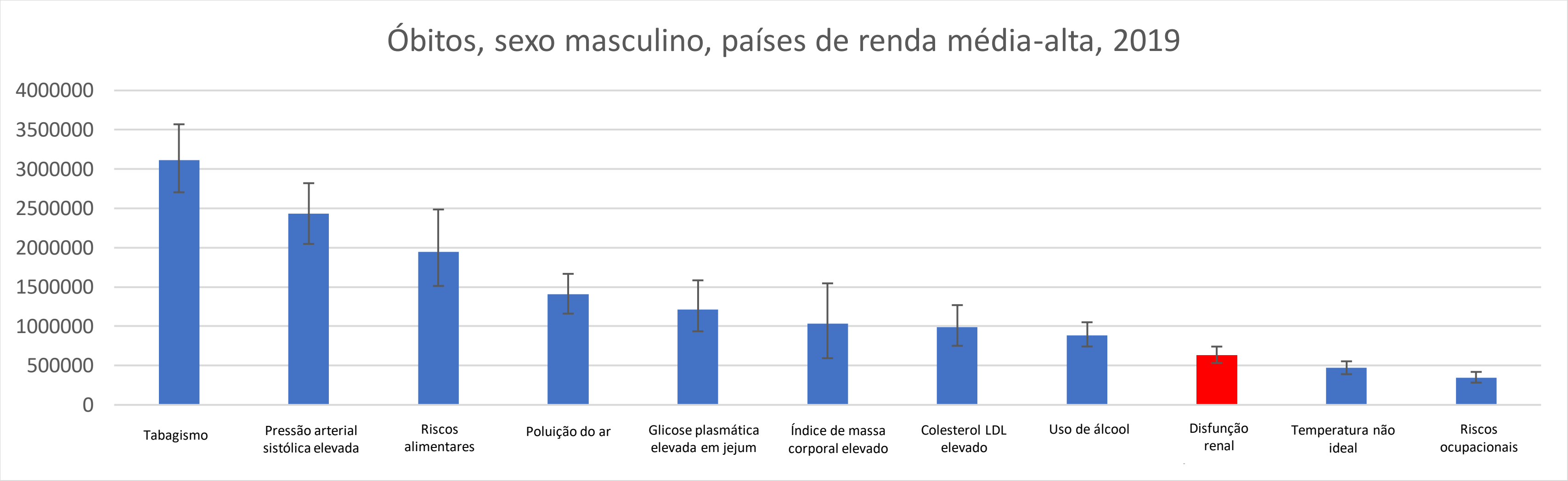
Óbitos, sexo masculino, países de renda média-alta, 2019
4000000
3500000
3000000
2500000
2000000
1500000
1000000
500000
0
Tabagismo
Pressão arterial sistólica elevada
Riscos alimentares
Poluição do ar
Glicose plasmática elevada em jejum
Índice de massa corporal elevado
Colesterol LDL elevado
Uso de álcool
Disfunção renal
Temperatura não ideal
Riscos ocupacionais

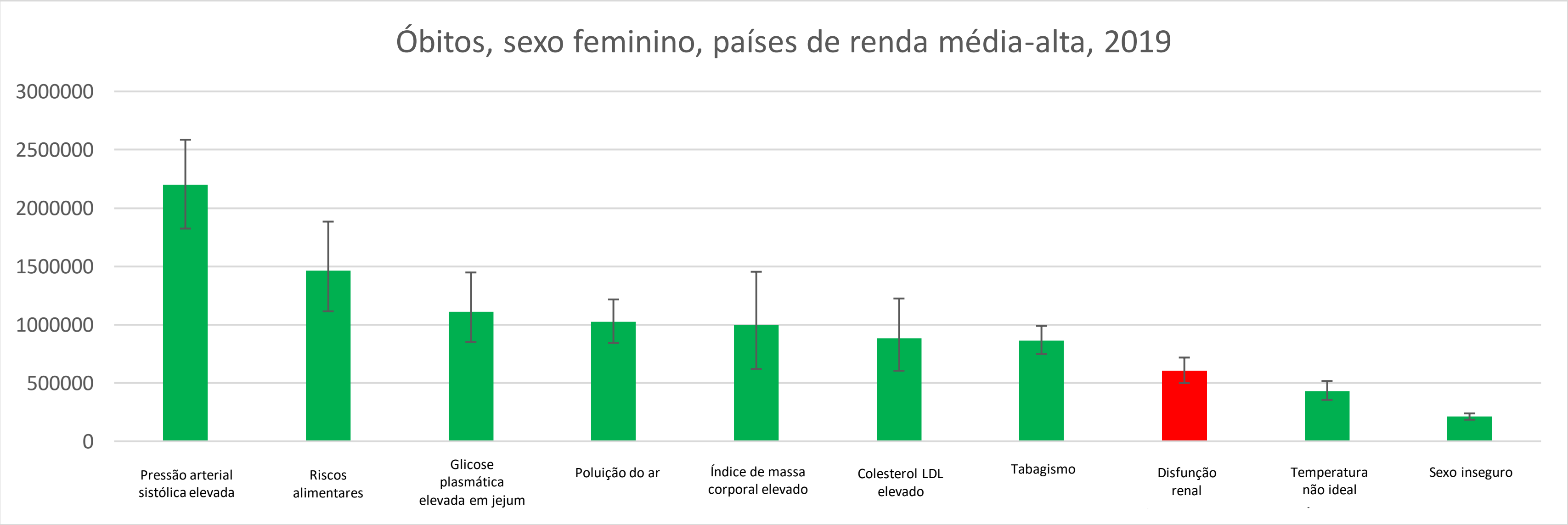
Óbitos, sexo feminino, países de renda média-alta, 2019
3000000
2500000
2000000
1500000
1000000
500000
0
Pressão arterial sistólica elevada
Riscos alimentares
Glicose plasmática elevada em jejum
Poluição do ar
Índice de massa corporal elevado
Colesterol LDL elevado
Tabagismo
Disfunção renal
Temperatura não ideal
Sexo inseguro

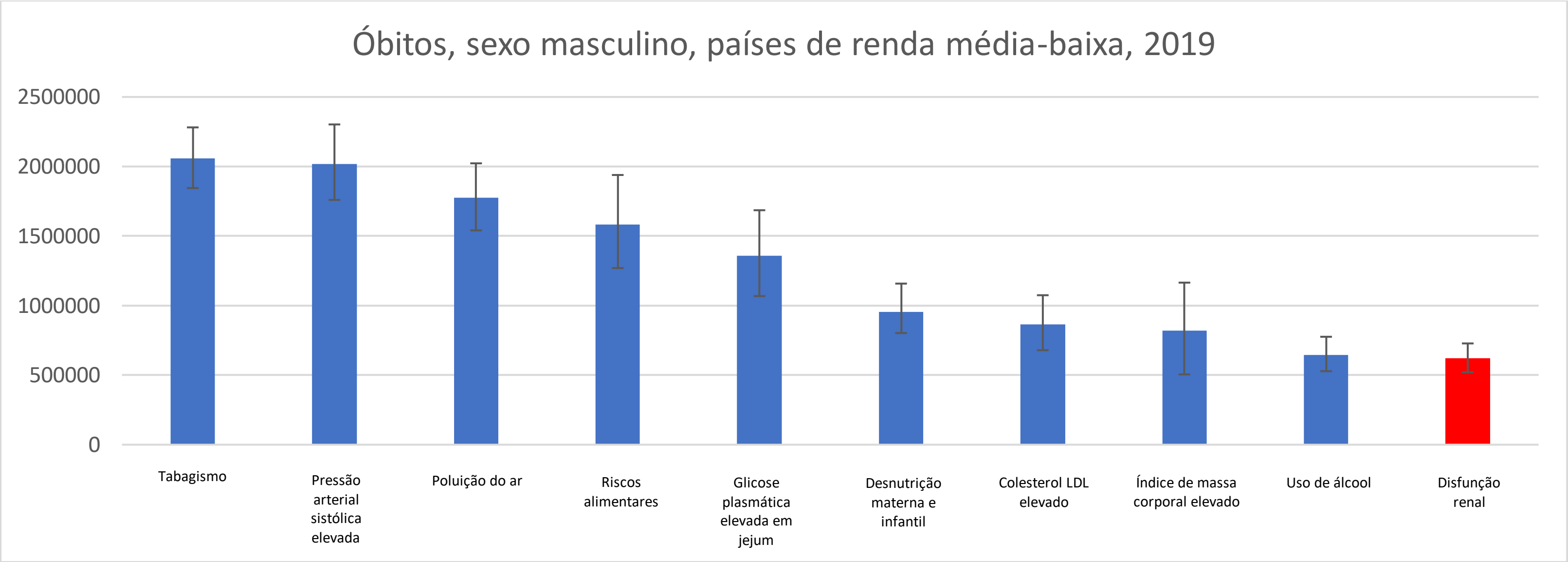
Óbitos, sexo masculino, países de renda média-baixa, 2019
2500000
2000000
1500000
1000000
500000
0
Tabagismo
Pressão arterial sistólica elevada
Poluição do ar
Riscos alimentares
Glicose plasmática elevada em jejum
Desnutrição materna e infantil
Colesterol LDL elevado
Índice de massa corporal elevado
Uso de álcool
Disfunção renal

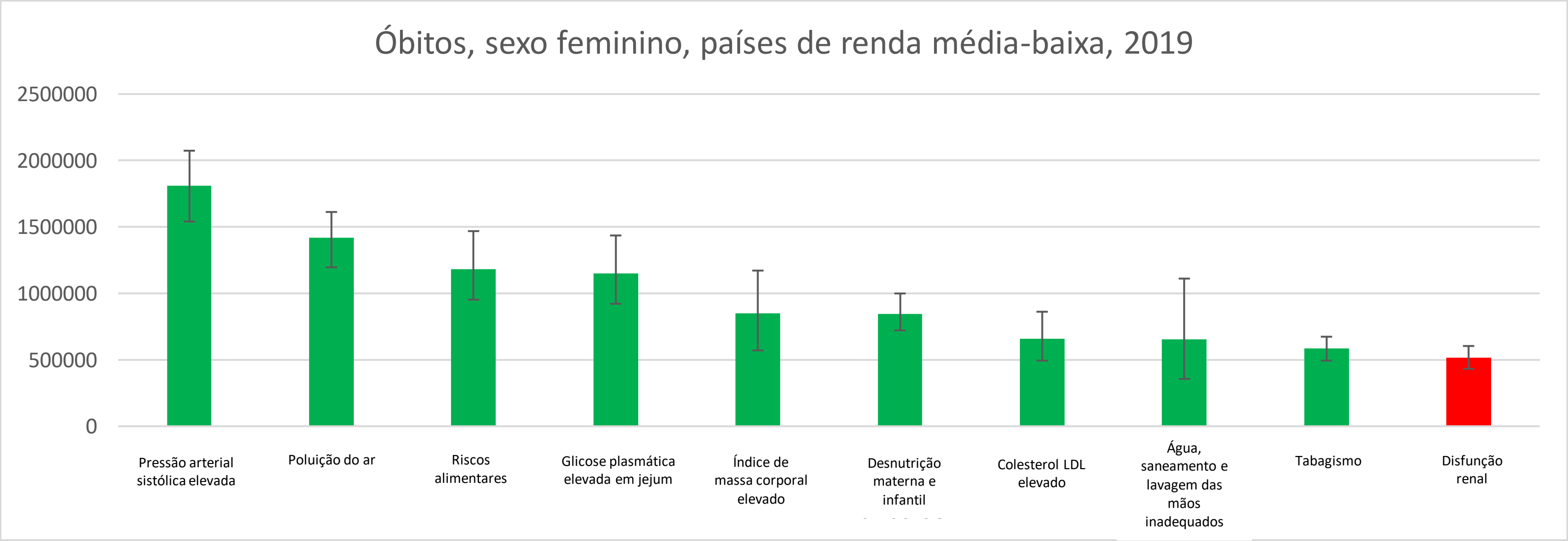
Óbitos, sexo feminino, países de renda média-baixa, 2019
2500000
2000000
1500000
1000000
500000
0
Pressão arterial sistólica elevada
Poluição do ar
Riscos alimentares
Glicose plasmática elevada em jejum
Índice de massa corporal elevado
Desnutrição materna e infantil
Colesterol LDL elevado
Água, saneamento e lavagem das mãos inadequados
Tabagismo
Disfunção renal

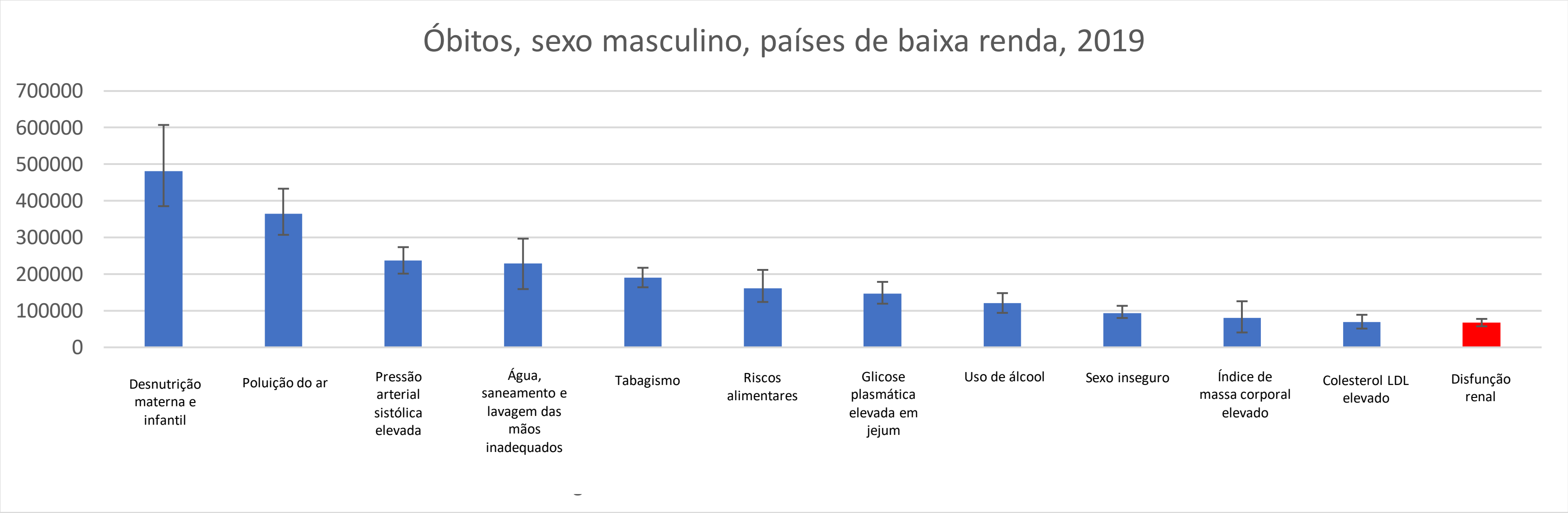

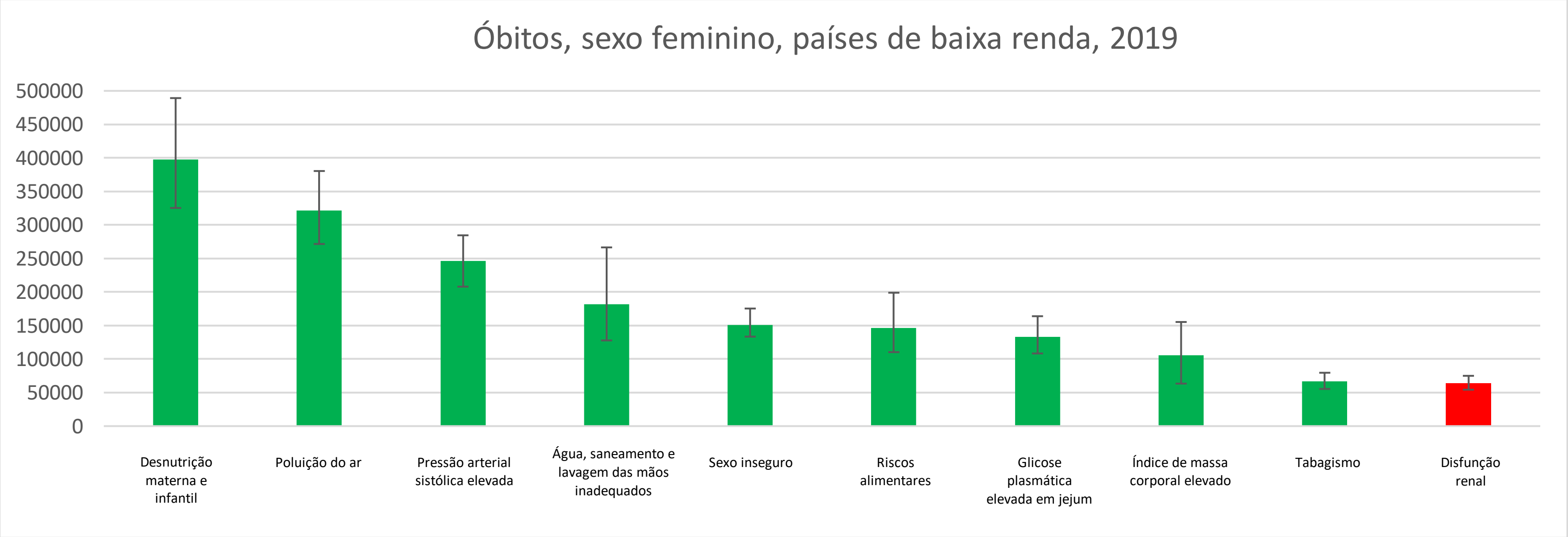

Dados do estudo *Global Burden of Diseases*: https://vizhub.healthdata.org/gbd-results/
