# Supplementary material for: Mind the gap in kidney care: translating what we know into what we do
Source: J Bras Nefrol. 2024 Jul 5;46(3):e2024E007. doi: 10.1590/2175-8239-JBN-2024-E007en (PMC11239182; doi:10.1590/2175-8239-JBN-2024-E007en)
Supplement: Supplementary file 5 [file 2175-8239-jbn-46-3-e2024E007-suppl5.pdf]

Braz. J. Nephrol. (J. Bras. Nefrol.) 2024, 46(3):e2024E007
https://doi.org/10.1590/2175-8239-JBN-2024-E007pt

Material Suplementar para “Atenção às lacunas no cuidado renal: traduzindo o que sabemos em ações”

TABELA S1 Novos agentes terapêuticos aprovados e emergentes para diferentes doenças renais.

**Medicamentos (Classe)**	**Condições**	**Status**	**Ensaios clínicos**
Inaxaplina (inibidor de APOL1)	GESF com duas variantes de APOL1	Emergente	NCT04340362
Sparsentan (antagonista do receptor da Endotelina A)	GESF	Emergente	DUPLEX (NCT03493685)
	NIgA	Aprovado	PROTECT (NCT03762850)
Budesonida (Corticosteroide)	NIgA	Aprovado	NEFIGARD (NCT03643965)
Atrasentan (antagonista do receptor da Endotelina A)	NIgA	Emergente	ALIGN (NCT04573478)
Sibeprenlimabe (inibidor de APRIL)	NIgA	Emergente	VISIONARY (NCT05248646)
Narsoplimabe (inibidor de MASP-2)	NIgA	Emergente	ARTEMIS-IgAN (NCT02682407)
Iptacopan (inibidor do fator B)	NIgA	Emergente	APPLAUSE-IgAN (NCT04578834)
	SHUa	Emergente	APPELHUS (NCT04889430)
Ravulizumabe (anticorpo monoclonal anti-C5 de ação prolongada)	SHUa	Aprovado	ALXN1210-aHUS311 (NCT02949128)
Dapagliflozina/ Empagliflozina (iSGLT2)	DRC/ DRD	Aprovado	DAPA-CKD (NCT03036150)<br>EMPA-kidney (NCT03594110)
Canagliflozina (iSGLT2)	DRD	Aprovado	CREDENCE (NCT02065791)
Finerenona (antagonistas dos receptores mineralocorticoides não esteroidais)	DRD	Aprovado	FIGARO-DKD (NCT02545049)<br>FIDELIO-DKD (NCT02540993)
	DRC	Emergente	FIND-CKD (NCT05047263)
Ziltivekimab (inibição de IL-6 mediada por anticorpo)	DRC	Emergente	RESCUE (NCT03926117)<br>RESCUE II (NCT04626505)
	DCV aterosclerótica na DRC		ZEUS (NCT05021835)
Semaglutida (agonista do receptor de GLP-1)	DRD	Emergente	FLOW (NCT03819153)

**Medicamentos (Classe)**	**Condições**	**Status**	**Ensaios clínicos**
Voclosporina (Inibidor da calcineurina)	Nefrite lúpica	Aprovado	AURORA1/2 (NCT03021499)
Belimumabe (anticorpo monoclonal anti-BAFF)	Nefrite lúpica	Aprovado	BLISS-LN (NCT01639339)
Anifrolumabe (anticorpo anti-interferon α)	Nefrite lúpica	Emergente	IRIS (NCT05138133)
Daratumumabe (anticorpo monoclonal anti-CD38)	Nefrite lúpica	Emergente	NCT04868838
Avacopan (antagonista do receptor C5a)	Vasculite por ANCA	Aprovado	ADVOCATE (NCT02994927)

SHUa, síndrome hemolítica urêmica atípica; DRC, doença renal crônica; DRD, doença renal diabética; GESF, glomeruloesclerose segmentar focal; NIgA, nefropatia por IgA
