# Supplementary material for: Mind the gap in kidney care: translating what we know into what we do
Source: J Bras Nefrol. 2024 Jul 5;46(3):e2024E007. doi: 10.1590/2175-8239-JBN-2024-E007en (PMC11239182; doi:10.1590/2175-8239-JBN-2024-E007en)
Supplement: Supplementary file 6 [file 2175-8239-jbn-46-3-e2024E007-suppl6.pdf]

Braz. J. Nephrol. (J. Bras. Nefrol.) 2024, 46(3):e2024E007
https://doi.org/10.1590/2175-8239-JBN-2024-E007pt

Material Suplementar para "Atenção às lacunas no cuidado renal: traduzindo o que sabemos em ações"

TABELA S2 Comentários dos pacientes sobre acessibilidade, viabilidade econômica, conhecimento, facilitadores e obstáculos ao tratamento renal ideal.

**Comentário/categoria do paciente**		**Custo**	**Efeitos colaterais, interações**	**Empoderamento do paciente**	**Segurança**	**Fonte de informações**	**Nível de conforto para questionamentos**	**Atraso na nova terapia**	**Nova esperança**	**Políticas/ Prevenção**	**Educação**	**Fragmentação do atendimento**	**Falta de especialistas**
**HK 1**	A medicação não é coberta pela CUS ou pelo seguro. Não posso me dar ao luxo de tomar os medicamentos mais eficazes conforme recomendado pelos meus médicos. Decido não tomar o medicamento, optar por uma alternativa mais barata ou começar a racionar a dose regular.	x											
**HK 2**	Considerando os efeitos colaterais, não tomar o medicamento na medida do possível seria a melhor política.		x										
**HK 3**	Não sinto nenhum efeito colateral e questiono a eficácia do medicamento.		x	x									
**HK 4**	Estou preocupado ou inseguro sobre as interações entre os medicamentos tomados, especialmente porque estou consultando médicos de diferentes especialidades, cada um deles prescrevendo seus próprios regimes separados.		x										
**HK 5**	Posso parar de tomar o medicamento quando meus resultados laboratoriais melhorarem ou quando eu começar a me sentir melhor.			x									
**HK 6**	Todos os medicamentos de venda livre geralmente são seguros para eu tomar.		x								x		
**HK 7**	A dose e as variedades de medicamentos continuam aumentando. Não tenho certeza se isso se deve à piora da condição ou à menor eficácia da medicação.		x										
**HK 8**	Sou cético quanto à adição de novos medicamentos ao meu regime atual e tenho a tendência natural de resistir a quaisquer novas adições ou aumento da dose.				x								
**HK 9**	Como paciente experiente, às vezes paro ou ajusto a dose dos medicamentos prescritos sem consultar meus médicos. Ou, se eles perguntarem, eu lhes digo que estou cumprindo totalmente o tratamento.			x									
**HK 10**	Meu conhecimento sobre medicamentos vem principalmente de um colega paciente que parece ser muito bem informado sobre esse assunto.					x							
**UK 1**	As pessoas precisam de confiança para contar aos médicos sobre os efeitos colaterais dos medicamentos, e os médicos podem oferecer educação e incentivo para explicar a medicação (e também incentivar as pessoas a não pararem de tomá-la sem discuti-la).		x				x						

Comentário/categoria do paciente		Custo	Efeitos colaterais, interações	Empoderamento do paciente	Segurança	Fonte de informações	Nível de conforto para questionamentos	Atraso na nova terapia	Nova esperança	Políticas/ Prevenção	Educação	Fragmentação do atendimento	Falta de especialistas
**UK 2**	Na Inglaterra, temos taxas de prescrição para determinados medicamentos, a menos que você esteja em diálise. Essas taxas, de cerca de £10 cada, podem ser uma barreira para alguns. No entanto, na Escócia, no País de Gales e na Irlanda do Norte não há cobranças.	x											
**UK 3**	Os novos medicamentos podem levar muito tempo para chegar aos pacientes, mesmo após a aprovação dos órgãos reguladores, pois os responsáveis pelo pagamento podem ficar relutantes.							x					
**UK 4**	Novos medicamentos, como os que podem retardar a DRC, foram recebidos com entusiasmo por muitos.								x				
**UK 5**	Um foco universal em abordagens preventivas para a DRC, incluindo exercícios, dieta, apoio emocional, além de medicamentos, é o que provavelmente será mais centrado no paciente, mas precisa de um plano.									x			
**HN 1**	Do ponto de vista dos pacientes renais em Honduras/América Latina, o acesso a medicamentos é crucial para nossa qualidade de vida e sobrevivência. Para isso, mencionamos alguns pontos de importância relacionados ao acesso a medicamentos e o que isso significa, de acordo com nossa experiência atual, com base no tema do Dia Mundial do Rim de 2024, "Saúde renal para todos: Promovendo o acesso equitativo aos cuidados e à prática ideal de medicação"	x											
**HN**	Na ausência de programas/políticas sólidas relacionadas ao manejo de doenças renais abrangentes, como em Honduras, existem programas isolados para o cuidado renal, em que a ação é voltada principalmente para o tratamento de diálise apenas. A responsabilidade do governo pela assistência pública cobre 85% da população renal, 12% a 13% é coberta pelo sistema de seguridade social e cerca de 3% pelo sistema privado. Atualmente, em Honduras, a legislação não cobre os interesses dos pacientes renais e deve ser atualizada e revisada para administrar o amplo programa de doenças renais.	x								x			
**HN**	Há uma diversidade significativa observada nos demais países da América Latina nas políticas de saúde relativas aos Programas de Saúde Renal promovidos pela SLANH. No entanto, muitos deles apresentam deficiências que vão desde o diagnóstico oportuno e precoce à falta de cobertura e acessibilidade aos medicamentos. Na América Latina, nos últimos anos, na maioria dos países, o foco na acessibilidade dos medicamentos tem sido no preço, em vez de no benefício, na eficácia e na qualidade dos medicamentos.	x								x			
**HN**	Em Honduras, o acesso a medicamentos é limitado devido a barreiras econômicas e ao tipo de sistema de saúde gerido. Muitos pacientes lutam para obter os medicamentos que necessitam, mas nem sempre é possível obtê-los e os custos para pacientes de baixa renda são inacessíveis. Embora o Ministério da Saúde tenha um conjunto básico de medicamentos, ele não atende às necessidades e não se ajusta ao crescimento da população renal, portanto, é insuficiente.	x											

Comentário/categoria do paciente		Custo	Efeitos colaterais, interações	Empoderamento do paciente	Segurança	Fonte de informações	Nível de conforto para questionamentos	Atraso na nova terapia	Nova esperança	Políticas/ Prevenção	Educação	Fragmentação do atendimento	Falta de especialistas
**HN**	Em Honduras, a ausência de programas definidos na atenção primária para a detecção precoce ou oportuna de doenças crônicas, como a renal, e a existência de um sistema de saúde bastante colapsado levaram ao aumento da doença renal na última década, o que tem sido agravado pela falta de orçamento para a saúde. Por essas razões, os pacientes não conseguem obter tratamentos adequados de qualidade e com os requisitos regulatórios exigidos. Por outro lado, a falta de recursos profissionais especializados nos afeta diretamente, uma vez que só há atendimento médico em casos de extrema urgência, fazendo com que, em muitos casos, já se detectem complicações muito avançadas.	x								x			
**HN**	Na América Latina, alguns países que desenvolveram uma cobertura nacional de saúde pública, como a Argentina e o Uruguai, não apresentam grandes déficits, mas o restante dos países tem muitas deficiências em seus sistemas públicos em termos de atendimento médico oportuno. Existe uma falta de educação em relação à doença para os pacientes.	x								x	x		
**HN**	Temos pouquíssimos profissionais de saúde especializados na área renal e a maioria está concentrada nas principais cidades do país em Honduras, com poucos ou nenhum nas demais áreas onde cresce o número de pacientes acometidos por doenças renais. Isso afeta diretamente o paciente, pois eles precisam migrar para as cidades para serem tratados, afetando sua qualidade de vida, ou têm que abandonar o tratamento por falta de recursos. Embora em Honduras, nos últimos anos, tenhamos tido um aumento no número de especialistas renais, eles se concentram em duas áreas do país, deixando descobertas as outras 16 áreas. São necessárias políticas para ajudar a melhorar essa escassez, a fim de melhorar o atendimento, especialmente nos hospitais da rede da Secretaria de Saúde. As barreiras de acessibilidade ao atendimento precisam ser rompidas.									x			x
**HN**	Em Honduras, nos últimos anos, conseguimos organizar alguns grupos ou ONGs de pacientes que lutam por melhorias na acessibilidade a medicamentos adequados e oportunos, com a qualidade dos mesmos de acordo com os padrões regulatórios necessários, mas isso representa apenas uma pequena porcentagem da população afetada. É por esse motivo que lutamos pela educação para o empoderamento dos pacientes. No entanto, frequentemente encontramos barreiras e paradigmas por parte das sociedades médicas e autoridades de saúde, mas temos conseguido e feito abordagens por escrito, bem como, pouco a pouco, conseguimos o envolvimento com entidades que lidam diretamente com a prestação de serviços e gestão de medicamentos.			x									

Comentário/categoria do paciente		Custo	Efeitos colaterais, interações	Empoderamento do paciente	Segurança	Fonte de informações	Nível de conforto para questionamentos	Atraso na nova terapia	Nova esperança	Políticas/ Prevenção	Educação	Fragmentação do atendimento	Falta de especialistas
**HN**	Em nível de América Latina, trabalhamos no empoderamento das diferentes ações necessárias para abordar os programas de saúde renal por meio de sociedades científicas em diferentes aspectos educacionais da perspectiva do paciente e dos profissionais de saúde, tentando fazer alianças de trabalho conjunto para aumentar a conscientização sobre as doenças subjacentes que podem levar à doença renal. Todo esse trabalho é realizado por meio da participação em redes de associações em nível nacional e internacional.			x							x		
**HN**	Em resumo, o acesso a medicamentos é essencial para pacientes renais em Honduras, pois esses medicamentos não apenas aliviam a dor e o desconforto, mas também são essenciais para prevenir complicações graves e manter uma qualidade de vida aceitável. Garantir o acesso equitativo a cuidados e medicamentos é essencial para atender às necessidades de saúde renal de todos os pacientes no país. E, ao mesmo tempo, por meio das diferentes ações e lutas que são executadas, nos capacita a poder participar das diferentes políticas de saúde necessárias para ter um atendimento médico digno e adequado, com acesso às melhores práticas de saúde por especialistas renais e aos medicamentos apropriados, eficazes e de qualidade em tempo hábil.	x											
**IN1**	Os pacientes são bem orientados sobre a necessidade de medicamentos em todos os estágios. Quando a prescrição muda, são necessárias mais informações sobre a necessidade de mudança para um melhor desfecho.										x		
**IN2**	É necessário estabelecer a correlação entre os medicamentos e as necessidades dietéticas.		x		x								
**IN3**	O custo é um fator importante tanto para pacientes de baixa quanto de média renda.	x											
**IN4**	Medicamentos excepcionais para doenças raras, como o eculizumabe, devem ser disponibilizados a custos razoáveis em todo o mundo.	x								x			
