# Supplementary material for: Mind the gap in kidney care: translating what we know into what we do
Source: J Bras Nefrol. 2024 Jul 5;46(3):e2024E007. doi: 10.1590/2175-8239-JBN-2024-E007en (PMC11239182; doi:10.1590/2175-8239-JBN-2024-E007en)
Supplement: Translation

# Translation: Atenção às lacunas no cuidado renal: traduzindo o que sabemos em ações

**DOI:** 10.1590/2175-8239-JBN-2024-E007pt

**Authors:** Valerie A. Luyckx, Katherine R. Tuttle, Dina Abdellatif, Ricardo Correa-Rotter, Winston W.S. Fung, Agnès Haris, Li-Li Hsiao, Makram Khalife, Latha A. Kumaraswami, Fiona Loud, Vasundhara Raghavan, Stefanos Roumeliotis, Marianella Sierra, Ifeoma Ulasi, Bill Wang, Siu-Fai Lui, Vassilios Liakopoulos, Alessandro Balducci, O Comitê Diretor Conjunto do Dia Mundial do Rim

**Affiliations:** 1 University of Zurich, Epidemiology, Biostatistics and Prevention Institute, Departmento de Saúde Pública e Global, Zurique, Suíça.; 2 Harvard Medical School, Brigham and Women’s Hospital, Departamento de Medicina, Divisão Renal, Boston, Massachusetts, EUA.; 3 University of Cape Town, Departamento de Pediatria e Saúde Infantil, Cidade do Cabo, África do Sul.; 4 Providence Inland Northwest Health, Providence Medical Research Center, Spokane, Washington, EUA.; 5 University of Washington, Departamento de Medicina, Divisão de Nefrologia, Seattle, Washington, EUA.; 6 Cairo University Hospital, Departamento de Nefrologia, Cairo, Egito.; 7 National Medical Science and Nutrition Institute Salvador Zubiran, Departamento de Nefrologia e Metabolismo Mineral, Cidade do México, México.; 8 University of Hong Kong, Prince of Wales Hospital, Departamento de Medicina e Terapêutica, The Chinese Shatin, Hong Kong, China.; 9 Péterfy Hospital, Departamento de Nefrologia, Budapeste, Hungria.; 10 ISN Patient Liaison Advisory Group, Bruxelas, Bélgica.; 11 Tamilnad Kidney Research (TANKER) Foundation, Chennai, Índia.; 12 Aristotle University of Thessaloniki, AHEPA University Hospital Medical School, 2º Departamento de Nefrologia, Thessaloniki, Grécia.; 13 University of Nigeria, College of Medicine, Departamento de Medicina, Ituku-Ozalla, Enugu, Nigéria.; 14 The Chinese University of Hong Kong, Jockey Club School of Public Health and Primary Care, Divisão de Sistema, Política e Gestão de Saúde, Hong Kong, China.; 15 Italian Kidney Foundation, Roma, Itália.

**Keywords:** Doença Renal Crônica, Equidade, Cuidados Renais, Saúde Pública, Dia Mundial do Rim

## Abstract

Historicamente, são necessários, em média, 17 anos para que novos tratamentos passem da evidência clínica para a prática diária. Considerando os tratamentos altamente eficazes disponíveis atualmente para prevenir ou retardar o início e a progressão da doença renal, esse período é demasiadamente longo. Agora é o momento de reduzir a lacuna entre o que sabemos e aquilo que fazemos. Existem diretrizes claras para a prevenção e o manejo dos fatores de risco comuns para doenças renais, como hipertensão e diabetes, mas apenas uma fração das pessoas com essas condições é diagnosticada mundialmente, e um número ainda menor recebe tratamento adequado. Da mesma forma, a grande maioria das pessoas que sofrem de doença renal não têm conhecimento de sua condição, pois ela costuma ser silenciosa nos estágios iniciais. Mesmo entre pacientes que foram diagnosticados, muitos não recebem tratamento adequado para a doença renal. Levando em consideração as graves consequências da progressão da doença renal, insuficiência renal ou óbito, é imperativo que os tratamentos sejam iniciados precocemente e de maneira adequada. As oportunidades para diagnosticar e tratar precocemente a doença renal devem ser maximizadas, começando no nível da atenção primária. Existem muitas barreiras sistemáticas, que vão desde o paciente até o médico, passando pelos sistemas de saúde e por fatores sociais. Para preservar e melhorar a saúde renal para todos em qualquer lugar, cada uma dessas barreiras deve ser reconhecida para que soluções sustentáveis sejam desenvolvidas e implementadas sem mais demora.

Pelo menos 1 em cada 10 pessoas em todo o mundo sofre de doença renal^1^. De acordo com o estudo *Global Burden of Disease*, em 2019, mais de 3,1 milhões de óbitos foram atribuídos à disfunção renal, tornando-a o sétimo principal fator de risco de óbito em todo o mundo (Figura 1 e Figura S1)^2^. No entanto, a mortalidade global por todas as doenças renais pode, na verdade, atingir entre 5 e 11 milhões de pessoas por ano se contabilizarmos também a estimativa de vidas perdidas devido à lesão renal aguda e à falta de acesso à terapia renal substitutiva para insuficiência renal (IR), especialmente em locais com recursos limitados^3^. Essas elevadas taxas globais de mortalidade refletem disparidades na prevenção, detecção precoce, diagnóstico e tratamento da doença renal crônica (DRC)^4^. As taxas de mortalidade por DRC são especialmente altas em algumas regiões, particularmente na América Latina Central e na Oceania (Ilhas do Pacífico Sul), indicando a necessidade urgente de ação^5^.

**Figura 1:** Todas as idades, 10 principais fatores de risco globais para óbito, 2019. A disfunção renal (definida como taxa de filtração glomerular estimada <60 mL/min por 1,73 m^2^ ou relação albumina/creatinina ≥30 mg/g) foi o sétimo principal fator de risco global de nível 3 para óbito em 2019. Os três principais fatores de risco globais para doenças renais, incluindo hipertensão, diabetes e sobrepeso/obesidade, também são os principais fatores de risco globais para óbito; portanto, são necessárias estratégias holísticas para abordar todos os fatores de risco simultaneamente. A classificação é representada por milhões se os óbitos forem atribuídos aos fatores de risco. As barras de erro representam o intervalo de confiança. A classificação global de disfunção renal estratificada por categoria de renda do Banco Mundial e por sexo é mostrada na Figura Suplementar S1. Dados obtidos do estudo *Global Burden of Disease* ^2^. Abreviações – IMC: índice de massa corporal; LDL: lipoproteína de baixa densidade.

A DRC também representa um significativo ônus econômico global, com custos que aumentam exponencialmente à medida que a DRC progride, não somente devido aos custos de diálise e transplante, mas também devido às múltiplas comorbidades e complicações que se acumulam ao longo do tempo^6,7^. Nos Estados Unidos, os gastos do Medicare com taxas por serviço para todos os beneficiários com DRC foram de US$ 86,1 bilhões em 2021 (22,6% do total de despesas)^8^. Não existem dados de muitos locais com recursos limitados, onde a maioria dos custos é paga com recursos próprios. Um estudo recente do Vietnã relatou que o custo da DRC por paciente foi superior ao produto interno bruto per capita^7^. Na Austrália, estima-se que o diagnóstico precoce e a prevenção da DRC podem economizar 10,2 bilhões de dólares para o sistema de saúde em 20 anos^9^.

Embora as causas da DRC variem regionalmente, os principais fatores de risco atribuíveis à população, para anos de vida ajustados por doenças relacionadas à DRC e padronizados por idade, foram hipertensão arterial (51,4%), nível elevado de glicose plasmática em jejum (30,9%) e índice de massa corporal elevado (26,5%)^10^. Estes são também os principais fatores de risco globais para óbito (Figura 1). Apenas 40% das pessoas com hipertensão e 60% daquelas com diabetes estão cientes de seu diagnóstico, e uma proporção muito menor recebe tratamento e alcança as metas estabelecidas^11,12^. Além disso, pelo menos 1 em cada 5 pessoas com hipertensão e 1 em cada 3 pessoas com diabetes também apresentam DRC^13^.

Grande parte da DRC pode ser prevenida por meio de estilos de vida saudáveis, prevenção e controle de fatores de risco, prevenção de lesão renal aguda, otimização da saúde materna e infantil, mitigação das alterações climáticas e abordagem dos determinantes sociais e estruturais da saúde^3^. No entanto, os benefícios de algumas dessas medidas poderão ser percebidos somente em gerações futuras. Nesse ínterim, o diagnóstico precoce e a estratificação de risco criam oportunidades para instituir terapias que retardem, interrompam ou até mesmo revertam a DRC^14^. De forma preocupante, a conscientização sobre a DRC foi surpreendentemente baixa entre indivíduos com disfunção renal, com ≈80% a 95% dos pacientes desconhecendo seu diagnóstico nas diversas regiões do mundo (Figura 2)^15–20^. Pessoas estão morrendo devido à perda de oportunidades em detectar precocemente a DRC e oferecer o tratamento ideal!

**Figura 2:** Proporção de pessoas com doença renal crônica (DRC) que têm conhecimento de seu diagnóstico e estão recebendo o cuidado adequado recomendado pelas diretrizes. A proporção de pessoas com DRC que estão cientes de seu diagnóstico varia em todo o mundo, com taxas que vão de 7% a 20%. À medida que o estágio da DRC piora, o conhecimento sobre a doença aumenta. Entre aqueles com diagnóstico de DRC, a proporção média de pacientes que recebem medicação adequada para retardar a progressão da DRC (inibidores do sistema renina-angiotensina-aldosterona [SRAA] e inibidores do cotransportador de sódio-glicose 2 [SGLT2]) está abaixo do ideal, assim como aqueles que atingem a pressão arterial alvo, controle do diabetes e orientação nutricional. As metas de tratamento descritas na figura seguem as diretrizes do KDIGO (*Kidney Disease: Improving Global Outcomes*) de 2012^15^. A maioria dos dados é proveniente de cenários com muitos recursos; essas proporções provavelmente são menores em ambientes com recursos limitados. Os dados são mostrados para proporções de pacientes que atingem pressão arterial <130/80 mm Hg. Dados compilados de estudos anteriores^15–20^. Abreviação – HbA1c: hemoglobina A1c.

Ainda mais importante, a DRC é um dos principais fatores de risco para doenças cardiovasculares e, à medida que a doença renal progride, a morte cardiovascular e a IR tornam-se riscos concorrentes^21^. De fato, os dados do estudo *Global Burden of Disease* de 2019 mostraram que mais pessoas morreram de doença cardiovascular atribuída à disfunção renal (1,7 milhão de pessoas) do que da própria DRC (1,4 milhão de pessoas)^2^. Portanto, o cuidado com a doença cardiovascular também deve ser uma prioridade para pessoas com DRC.

## Lacunas Entre o Conhecimento e a Implementação no Cuidado Renal

As estratégias para prevenir e tratar a DRC foram desenvolvidas com base em sólidas evidências ao longo das últimas três décadas (Figura 3)^19,22^. As diretrizes de prática clínica para a DRC são claras; entretanto, a adesão a essas diretrizes está abaixo do ideal (Figura 2)^15,19,20^.

**Figura 3:** Manejo terapêutico e estilo de vida ideais recomendados para a doença renal crônica (DRC) no diabetes. Ilustração de uma abordagem abrangente e holística para a saúde renal ideal em pessoas com DRC. Além dos ajustes fundamentais no estilo de vida, a atenção ao controle do diabetes, da pressão arterial (PA) e dos fatores de risco cardiovascular é essencial para o cuidado com os rins.

Abreviações – DCVA: doença cardiovascular aterosclerótica; DMO-DRC: distúrbio mineral ósseo na doença renal crônica; TFGe: taxa de filtração glomerular estimada; AR GLP-1: agonista do receptor de peptídeo-1 semelhante ao glucagon; HTN: hipertensão; ARM: antagonistas do receptor mineralocorticoide; ARM-ne: antagonistas do receptor mineralocorticoide não esteroidal; iPCSK9: inibidor da proproteína convertase subtilisina/kexina tipo 9; SRAA: sistema renina-angiotensina-aldosterona; PAS: pressão arterial sistólica; iSGLT2: inibidor do cotransportador de sódio-glicose 2. Notas – *O inibidor da enzima conversora da angiotensina ou os bloqueadores dos receptores da angiotensina II devem ser a terapia de primeira linha para o controle da PA na presença de albuminúria; caso contrário, um bloqueador dos canais de cálcio diidropiridínico (BCC) ou um diurético também podem ser considerados.

Figura reproduzida a partir do Grupo de Trabalho KDIGO (*Kidney Disease: Improving Global Outcomes*) para DRC. Diretriz de Prática Clínica KDIGO 2024 para Avaliação e Manejo da Doença Renal Crônica. *Kidney Int*. https://doi.org/10.1016/j.kint.2023.10.018 ^22^. Copyright^©^ 2023, *Kidney Disease*: *Improving Global Outcomes* (KDIGO). Publicado pela Elsevier Inc. em nome da Sociedade Internacional de Nefrologia sob a licença CC BY-NC-ND (https://creativecommons.org/licenses/by-nc-nd/4.0/).

Independentemente da causa, o controle dos principais fatores de risco, especialmente diabetes e hipertensão, é a base do tratamento ideal para a DRC^19,23^. Além das alterações no estilo de vida e controle dos fatores de risco, as classes farmacológicas iniciais de agentes que comprovadamente proporcionam proteção renal foram os inibidores do sistema renina-angiotensina-aldosterona na forma de inibidores da enzima conversora da angiotensina (IECAs) e os bloqueadores dos receptores de angiotensina^14,19^. Apesar de serem conhecidos há décadas por terem efeitos protetores importantes sobre a função renal e cardíaca em pessoas com DRC, o uso desses medicamentos permanece baixo com base em dados do mundo real extraídos de registros eletrônicos de saúde (Figura 2). Por exemplo, nos Estados Unidos, foi relatado que o uso de IECA ou bloqueadores dos receptores de angiotensina varia de 20% a 40% mais de 15 anos após sua última aprovação para pacientes com DRC e diabetes tipo 2^24^. Embora dados mais recentes mostrem que as taxas de prescrição melhoraram para 70% nessa população, somente 40% permanecem em uso de um IECA ou bloqueador dos receptores de angiotensina por pelo menos 90 dias^20^. Esses dados ilustram lacunas tanto na prescrição de medicamentos protetores renais quanto na continuidade dos cuidados ao longo do tempo, potencialmente relacionadas ao custo, falta de instrução do paciente, polifarmácia e efeitos adversos^25^.

Embora o entusiasmo inicial pelos inibidores do cotransportador de sódio-glicose-2 (SGLT2) tenha se concentrado em seus benefícios para diabetes e doenças cardiovasculares, benefícios terapêuticos sem precedentes foram claramente observados também para a DRC. As reduções de risco relativo com os inibidores do SGLT2 se aproximam de 40% para um declínio substancial na taxa de filtração glomerular estimada, IR e óbito em populações com DRC de diversas causas, insuficiência cardíaca ou alto risco de doença cardiovascular^26,27^. Esses benefícios foram observados em adição ao manejo padrão dos fatores de risco e ao uso de inibidores do sistema renina-angiotensina-aldosterona. Os riscos de insuficiência cardíaca, morte cardiovascular e mortalidade por todas as causas também foram reduzidos em pacientes com DRC^26^. A adição de inibidores do SGLT2 aos inibidores do sistema renina-angiotensina-aldosterona pode retardar a necessidade de terapia renal substitutiva por vários anos, dependendo do momento em que são iniciados^28^. Além disso, para cada 1.000 pacientes com DRC tratados com um inibidor do SGLT2 além da terapia padrão, é possível evitar 83 óbitos, 19 hospitalizações por insuficiência cardíaca, 51 iniciações de diálise e 39 episódios de piora aguda da função renal^29^.

É preocupante o fato de ainda haver uma subutilização acentuada dessas e de outras terapias recomendadas pelas diretrizes, incluindo os inibidores do SGLT2 (Figura 2)^20,24^. No registro CURE-CKD, apenas 5% e 6,3% dos pacientes elegíveis com DRC e diabetes, respectivamente, continuaram sob o uso de inibidores do SGLT2 e agonistas do receptor do peptídeo-1 semelhante ao glucagon após 90 dias^18^. Notavelmente, a falta de seguro saúde comercial e o tratamento em instituições comunitárias versus instituições acadêmicas foram associados a menores probabilidades de prescrição de inibidores do SGLT2, IECAs ou bloqueadores dos receptores de angiotensina entre pacientes com diabetes e DRC^20^. Em países de baixa ou média renda (PBMR), a lacuna entre as evidências e a implementação é ainda maior, devido ao custo elevado e à disponibilidade inconsistente desses medicamentos, apesar da disponibilidade de genéricos^30^. Essas lacunas no fornecimento do tratamento ideal para a DRC são inaceitáveis.

Além dos inibidores do SGLT2, demonstrou-se que os antagonistas dos receptores mineralocorticoides não esteroidais, somados ao tratamento padrão com inibidores do sistema renina-angiotensina-aldosterona, reduzem os riscos de progressão da DRC, IR, eventos cardiovasculares e óbitos no diabetes tipo 2^31^. Um portfólio crescente de opções terapêuticas promissoras está surgindo no horizonte, com agonistas do receptor do peptídeo-1 semelhante ao glucagon (NCT03819153, NCT04865770), inibidores da aldosterona sintase (NCT05182840) e incretinas duplas a triplas (Tabela S1)^26,32^. Além disso, já existem evidências claras de que, em pacientes com DRC e diabetes, os agonistas do receptor do peptídeo 1 semelhante ao glucagon reduzem os eventos cardiovasculares, são terapias seguras e eficazes para redução da glicose e auxiliam na perda de peso^32^.

Historicamente, leva-se, em média, 17 anos para que novos tratamentos passem da evidência clínica para a prática diária^33^. Com milhões de pessoas com DRC morrendo a cada ano, essa é uma espera muito longa.

## Fechando a “Lacuna” Entre o que Sabemos e o Que Fazemos

### Ausência de Políticas, Desigualdades Globais

#### Política de Saúde

Desde o lançamento do Plano de Ação da Organização Mundial da Saúde para Doenças Não Transmissíveis (DNTs) em 2013, houve progresso global na proporção de países com um plano de ação nacional para DNTs e unidades dedicadas a essas doenças^34^. No entanto, a DRC só é incorporada às estratégias de DNTs em aproximadamente metade dos países^4^. São necessárias políticas para integrar os cuidados renais aos pacotes essenciais de saúde no âmbito da cobertura universal de saúde (Figura 4)^30^. As políticas multissetoriais também devem abordar os determinantes sociais de saúde, que aumentam significativamente o risco e a gravidade da DRC, limitando as oportunidades das pessoas em melhorar sua saúde^3^. A falta de investimento na promoção da saúde renal, juntamente com as prevenções primária e secundária da doença renal, prejudica o progresso^14^.

**Figura 4:** Representação do espectro de fatores que afetam a implementação de cuidados renais oportunos e de qualidade.

Abreviações – DRC: doença renal crônica; DNT: doenças não transmissíveis; CUS: cobertura universal de saúde.

#### Sistemas de saúde

Dois dos principais objetivos da cobertura universal de saúde são a cobertura de serviços essenciais de saúde e a redução das dificuldades financeiras impostas pela assistência médica. No entanto, a cobertura universal de saúde por si só não é suficiente para garantir acesso adequado ao tratamento renal^3^. Os sistemas de saúde devem ser fortalecidos e a qualidade do atendimento deve ser priorizada, uma vez que o atendimento de baixa qualidade contribui para mais óbitos do que a falta de acesso em ambientes com recursos limitados^35^. O atendimento de qualidade requer uma força de trabalho em saúde bem treinada, disponibilidade sustentável de diagnósticos precisos, infraestrutura confiável e suprimentos de medicamentos, e deve ser monitorado em um processo contínuo de melhoria da qualidade (Figura 4). A qualidade dos medicamentos, especialmente em PBMRs, pode ser uma barreira adicional para o manejo bem-sucedido da DRC^36^. A regulamentação e o monitoramento da fabricação de medicamentos e dos padrões de qualidade são importantes para garantir terapias seguras e eficazes. Estratégias para apoiar a regulamentação e a garantia de qualidade deverão ser desenvolvidas de acordo com o contexto e orientações locais, conforme descrito em outras fontes^37^.

Estabelecer um caso confiável para detecção e manejo da DRC com base em riscos, intervenções e desfechos, além de custos com base em dados do mundo real, ajudará a traduzir a relação custo-benefício teórica (atualmente estabelecida principalmente em países de alta renda e com dados mínimos de outros países) dentro da realidade econômica^30,38^. A triagem deve incluir avaliação de fatores de risco para DRC, considerando histórico familiar, reconhecendo sintomas potenciais (geralmente fadiga avançada, falta de apetite, edema, coceira etc.) e medindo pressão arterial, creatinina sérica, urinálise e taxas de albumina/proteína/creatinina urinária, conforme descrito nas diretrizes estabelecidas^19,39^. Abordar a DRC desde o início, já na atenção primária, deve reduzir os custos ao longo do tempo, diminuindo as complicações da DRC e a IR. Os medicamentos necessários para tratamento renal já estão incluídos na Lista de Medicamentos Essenciais da Organização Mundial da Saúde (Tabela 1). Tais medicamentos devem ser fornecidos em nível nacional sob cobertura universal de saúde^40^. As empresas farmacêuticas devem disponibilizá-los a preços acessíveis.

**Tabela 1:** Medicamentos essenciais para pacientes com doença renal

Medicamento/tecnologia	Exemplo	Motivo	Na lista modelo de medicamentos essenciais da OMS
Inibidor da ECA	Enalapril, lisinopril	Retarda progressão da DRC, beneficia doença cardiovascular e acidente vascular cerebral	Sim
Bloqueador dos receptores da angiotensina	Losartana, telmisartana	Retarda progressão da DRC, doença cardiovascular e acidente vascular cerebral	Sim
Bloqueador dos canais de cálcio	Amlodipina, verapamil	Controle da pressão arterial	Sim
Diuréticos de alça	Furosemida, torsemida	Bom quando TFG está baixa, bom para insuficiência cardíaca	Sim
Diuréticos tiazídicos	Hidroclorotiazida, metolazona, indapamida	Bom para PA, especialmente na população negra	Sim
Inibidor do SGLT2	Empagliflozina, canagliflozina, dapagliflozina	Controle do diabetes, retarda progressão da DRC, doença cardiovascular e óbito	Sim
Agonista de GLP1	Semaglutida	Controle do diabetes, perda de peso	Não
Inibidor de mineralocorticoides	Espironolactona, finerenona	Atrasa progressão da DRC, reduz risco de insuficiência cardíaca Cuidado: risco de hipercalemia em pacientes com doença renal	Sim/não
β-Bloqueador	Bisoprolol	Prevenção e tratamento de isquemia cardíaca	Sim
Estatinas	Sinvastatina	Prevenção de DAC em pacientes com DRC, transplante	Sim
Aspirina		Prevenção secundária de IM em pacientes com DRC, transplante	Sim
Combinações de dose fixa (polipílula)^a^	Aspirina + atorvastatina + ramipril	Manejo simultâneo de DRC, doença cardiovascular e fatores de risco, quando indicado^a^	Sim
	Aspirina + sinvastatina + ramipril + atenolol + hidroclortiazida		Sim
	Aspirina + perindopril + amlodipina		Sim
Medicamento hipoglicemiante oral	Gliclazida, metformina, inibidores do SGLT2	Manejo de DM Cuidado com dosagem e taxa de filtração glomerular	Sim
Insulina	Ação longa e curta	Manejo de DM	Sim

Abreviações – ECA: enzima conversora da angiotensina; PA: pressão arterial; DAC: doença arterial coronariana; DRC: doença renal crônica; DM: diabetes mellitus; TFG: taxa de filtração glomerular; GLP1: peptídeo-1 semelhante ao glucagon; IM: infarto do miocárdio; SGLT2: cotransportador de sódio-glicose 2; OMS: Organização Mundial da Saúde.

Nota –

a Polipílulas contendo aspirina podem não ser apropriadas para pacientes com DRC inicial sem outras indicações cardiovasculares.

### Desafios na Atenção Primária, Inércia Clínica

#### Profissionais da saúde

A escassez de profissionais de cuidados primários é exacerbada pelo acesso inconsistente a especialistas e profissionais da saúde aliados, tanto em países de alta renda quanto em PBMRs. É essencial definir papéis e responsabilidades para o cuidado renal. As soluções podem incluir atendimento por equipe multidisciplinar (médicos de atenção primária, farmacêuticos, profissionais avançados, enfermeiros, terapeutas, educadores, nutricionistas e profissionais de saúde mental) com mecanismos bem estabelecidos de colaboração entre todos os elementos, além de tecnologias de comunicação prontamente disponíveis nos sistemas de saúde e entre profissionais para apoiar o cuidado e a tomada de decisões^41,42^. A fuga de cérebros em ambientes com recursos limitados é complexa e deve ser enfrentada.

A mobilização de agentes comunitários de saúde resulta em economia de custos nos programas de doenças infecciosas em PBMRs e pode facilitar a detecção precoce, o diagnóstico e o manejo de DNTs^43^. O manejo protocolizado da DRC, possivelmente suportado por sistemas eletrônicos de apoio à decisão, é adequado para intervenções em nível comunitário, com a integração de médicos da atenção primária e o apoio da nefrologia e de outros profissionais^44,45^. Em alguns ambientes, os farmacêuticos podem identificar pessoas com diabetes ou hipertensão em risco de DRC, com base em suas prescrições, e fornecer testes no local e encaminhamento, se necessário^46^. Os farmacêuticos também podem fornecer reconciliação medicamentosa e orientação sobre segurança, eficácia e adesão. Assistentes sociais e farmacêuticos podem auxiliar pacientes com programas de acesso a medicamentos^46^.

### Desafios Para a Inércia Clínica

A “inércia” clínica comumente responsabilizada pelas baixas taxas de prescrição apresenta diversas facetas (Figura 4)^47^. Existem muitas lacunas de conhecimento sobre a DRC entre os médicos da atenção primária^48^. Essas lacunas podem ser resolvidas com educação pública e profissional direcionada. Outros fatores incluem o medo de efeitos adversos dos medicamentos, incentivos desalinhados dentro do sistema de saúde, carga de trabalho excessiva, restrições de formulários e esgotamento dos médicos^47^. Além disso, discrepâncias nas recomendações das diretrizes de diferentes organizações profissionais podem contribuir para a confusão. Um dos principais impedimentos ao cuidado ideal são as restrições de tempo impostas aos médicos individuais. Em média, um profissional de atenção primária nos Estados Unidos precisaria de ≈26,7 horas por dia para implementar o atendimento recomendado pelas diretrizes para um painel de 2.500 pacientes^49^. A inovação é necessária para apoiar a implementação de diretrizes, especialmente para profissionais da atenção primária que devem implementar muitas diretrizes diferentes para atender às necessidades de vários pacientes. Registros eletrônicos de saúde, lembretes, incentivos baseados em equipes e ferramentas de apoio à decisão oferecem um suporte promissor para o cuidado renal de qualidade em práticas clínicas movimentadas^50^. O tempo e esforço extras gastos na negociação de pré-autorizações ou no preenchimento de solicitações de programas de assistência medicamentosa, juntamente com a necessidade de monitoramento frequente de múltiplos medicamentos, também dificultam a prescrição adequada^25^. Muitos profissionais da atenção primária têm apenas alguns minutos alocados por paciente devido à pressão institucional ou ao volume de pacientes. A “nércia” dificilmente pode ser aplicada a médicos que trabalham nesse ritmo. O número de profissionais de saúde deve aumentar mundialmente.

As consultas para pacientes com DRC são complexas, já que a multimorbidade é elevada. Os pacientes geralmente são tratados por diversos especialistas, o que leva à fragmentação do atendimento, falta de supervisão holística e difusão da responsabilidade pelo tratamento. O cuidado multidisciplinar melhorou a transição para a terapia renal substitutiva e reduziu a mortalidade em análises de desfechos únicos e combinados^51^. Novos modelos de “clínicas combinadas” com colaboração e coparticipação no local (nefrologista-cardiologista-endocrinologista) podem se revelar altamente benéficos para os pacientes, ao reduzir a fragmentação do atendimento, melhorar a logística e poupar custos.

### Centralização no Paciente

#### Alfabetização em saúde

O autocuidado é o aspecto mais importante do cuidado renal. A capacidade do paciente de compreender suas necessidades de saúde, fazer escolhas saudáveis, sentir-se seguro e respeitado no sistema de saúde e o apoio psicossocial são importantes para promover a tomada de decisões relativas à saúde (Figura 4). Uma boa comunicação requer informações de qualidade e, sobretudo, a confirmação do “entendimento” por parte do paciente e, muitas vezes, da família. Aplicativos eletrônicos e lembretes podem ser ferramentas úteis para apoiar os pacientes, melhorando o conhecimento sobre a doença, promovendo o empoderamento do paciente e melhorando a autoeficácia, embora seja improvável que “um modelo único sirva para todos”^52^. Informações insuficientes sobre a saúde do paciente, comunicação precária e falta de confiança, entre outros elementos, são barreiras importantes, especialmente em comunidades marginalizadas e minoritárias, onde a DRC é comum^30^. Os pacientes também podem ficar confusos com recomendações de cuidados contraditórias entre profissionais de saúde, bem como com mensagens conflitantes na mídia leiga. Plataformas inovadoras para melhorar a comunicação entre pacientes e médicos sobre a DRC são promissoras e podem promover a prescrição e adesão ideais^53,54^.

Para superar barreiras e promover a equidade, as perspectivas dos pacientes são essenciais para desenvolver e testar melhores estratégias de saúde. Os modelos de cuidado colaborativo devem incluir pacientes, famílias, grupos comunitários, diversos profissionais de saúde, sistemas de saúde, órgãos governamentais e contribuintes^38^. Organizações de defesa de direitos, grupos comunitários locais e navegadores de pares, que possuem vozes e relacionamentos confiáveis, podem ajudar a educar e fornecer contribuições para o desenvolvimento de ferramentas para pacientes e programas de alcance^55^. Sobretudo, os pacientes devem estar no centro de seus cuidados.

#### Custo e disponibilidade de medicamentos

Em países de alta renda, as pessoas sem seguro de saúde e aquelas com coparticipações elevadas, paradoxalmente, pagam mais caro até mesmo por medicamentos essenciais^38^. Nos PBMRs, a doença renal é a principal causa de gastos catastróficos com saúde devido à dependência de pagamentos diretos^56^. Em 18 países, 4 medicamentos para doença cardiovascular (estatinas, IECAs, aspirina e *β*-bloqueadores), todos frequentemente indicados na DRC, estavam mais disponíveis em ambientes privados do que públicos, na sua maioria indisponíveis em comunidades rurais e inacessíveis para 25% das pessoas em países de renda média-alta e 60% das pessoas em países de baixa renda^57^. Terapias mais recentes podem ser proibitivamente caras em todo o mundo, especialmente onde os genéricos podem ainda não estar disponíveis. Nos Estados Unidos, o preço de varejo para suprimento de um mês de um inibidor do SGLT2 ou finerenona é de ≈US$ 500 a US$ 700; e para agonistas do receptor do peptídeo-1 semelhante ao glucagon, de ≈US$ 800 a US$ 1.200 por mês^38^. A dependência do pagamento direto de medicamentos básicos vitais e que salvam vidas é inaceitável (Figura 4).

### Considerações Especiais

Nem todas as doenças renais são iguais. Muito do que foi discutido aqui está relacionado às formas mais comuns de DRC (por exemplo, diabetes e hipertensão). Algumas formas de DRC ainda não completamente compreendidas apresentam perfis de risco diferentes, incluindo exposições ambientais, predisposição genética e distúrbios autoimunes ou outras doenças sistêmicas. Podem ser necessárias terapias altamente especializadas. As empresas farmacêuticas devem ser responsáveis por garantir que os estudos de investigação incluam participantes representativos da doença, com representação adequada de raça, etnia, sexo e gênero, que medicamentos eficazes sejam disponibilizados após os estudos e que o equilíbrio entre lucros e preços seja justo e transparente. Muitas terapias novas oferecem uma nova esperança para diversas doenças renais e, uma vez aprovadas, não deve haver atrasos na extensão dos benefícios a todos os pacientes afetados (Tabela S1).

Um grupo importante, muitas vezes negligenciado, é o de crianças com doenças renais. Esse grupo é especialmente vulnerável em PBMRs, onde os serviços de nefrologia e os recursos são limitados, e as famílias muitas vezes precisam escolher entre pagar pelo tratamento de uma criança ou sustentar o restante da família^58^. As crianças com DRC também apresentam alto risco de doenças cardiovasculares, mesmo em ambientes de alta renda, e é necessária mais atenção para controlar os fatores de risco e atingir as metas de tratamento^59^.

## Promovendo a Inovação

### Ciência da implementação e tradução do conhecimento

Uma vez que sabemos como tratar a DRC a partir de uma rigorosa base de evidências, devemos agora otimizar a implementação^60^. A pesquisa de implementação tem como objetivo identificar soluções eficazes ao compreender como as práticas baseadas em evidências, geralmente desenvolvidas em países de alta renda, podem ser integradas às vias de cuidado em ambientes com recursos mais limitados. O manejo da DRC se presta à pesquisa de implementação: estratégias terapêuticas ideais são conhecidas, desfechos são facilmente mensuráveis e diagnósticos e medicamentos essenciais já devem estar em vigor. Obter as preferências dos pacientes locais e compreender os desafios são componentes cruciais dessa pesquisa. Os Ministérios da Saúde devem se comprometer a superar as barreiras identificadas e a ampliar os programas bem-sucedidos e sustentáveis.

### Polipílulas como exemplo de inovação simples

As polipílulas são atrativas em vários níveis: doses fixas de diversos medicamentos recomendados pelas diretrizes estão presentes em um único comprimido (Tabela 1), o preço é mais baixo, a carga de comprimidos é menor e o regime é simples^61^. As polipílulas demonstraram prevenir doenças cardiovasculares e são economicamente viáveis para pacientes com DRC^62^. São necessários mais estudos, mas, considerando as alternativas de terapia renal substitutiva dispendiosa ou óbito precoce, é provável que as polipílulas se mostrem uma opção de custo-benefício para reduzir a progressão da DRC.

### Aproveitando tecnologias digitais

A integração da telemedicina e outros tipos de cuidados prestados remotamente pode aumentar a eficiência e reduzir custos^63^. Registros e prontuários eletrônicos de saúde podem apoiar o monitoramento da qualidade do atendimento, identificar lacunas para orientar a implementação e melhorar desfechos nos sistemas de aprendizado em saúde. A inteligência artificial também pode ser utilizada para estratificação de risco e personalização da prescrição e adesão de medicamentos^64^. O uso da telenefrologia para comunicação entre a atenção primária e subespecialistas também pode se mostrar útil e benéfico para o tratamento do paciente^65^.

## Perspectivas dos Pacientes

Existem diversos métodos para obter as preferências dos pacientes para o cuidado da DRC, incluindo entrevistas, grupos focais, levantamentos, experimentos de escolha discreta, ferramentas estruturadas e simples conversas^66,67^. Atualmente, muitos desses métodos estão em fase de pesquisa. A tradução para a clínica demandará contextualização e determinação da aceitabilidade local e individual.

A jornada de cada pessoa que vive com DRC é única, mas está associada a desafios e barreiras. Como exemplos de experiências vividas, os comentários de pacientes sobre seus medicamentos e cuidados estão descritos no Quadro 1 e na Tabela S2. Essas vozes devem ser ouvidas e seguidas para eliminar lacunas e melhorar a qualidade do atendimento renal por toda parte.

**Quadro 1:** Barreiras que impactam o uso de medicamentos, conforme expressado por pessoas que sofrem de doença renal

“Tenho que pagar pelos meus medicamentos, então opto por alternativas mais baratas ou raciono a dose regular.” “Estou consultando médicos de diferentes especialidades, cada um deles prescrevendo regimes separados, o que me deixa preocupado com as interações medicamentosas.” “Como paciente experiente, às vezes paro ou modifico a dose dos medicamentos prescritos sem consultar meus médicos. Se eles perguntarem, eu lhes direi que estou seguindo totalmente o tratamento.” “Com o tempo, a dose e as variedades dos meus medicamentos continuam aumentando. Não tenho certeza se isso se deve à piora da condição ou se os medicamentos estão se tornando menos eficazes.” “Meu conhecimento sobre medicamentos vem principalmente de um colega paciente que parece ser muito bem informado sobre esse assunto.”

## Chamado à Ação

Um impasse no cuidado renal tem sido tolerado há tempo demais. Os novos avanços terapêuticos oferecem uma esperança real de que muitas pessoas com DRC possam sobreviver sem desenvolver IR. As evidências de benefícios clínicos são esmagadoras e inequívocas. Não podemos esperar mais 17 anos para que essas evidências sejam traduzidas em práticas clínicas^33^. Agora é o momento de garantir que todos os que são elegíveis para receber tratamento para DRC o recebam de forma equitativa.

As barreiras conhecidas e as disparidades globais no acesso ao diagnóstico e ao tratamento devem ser abordadas com urgência (Figura 4). Para alcançar a equidade em saúde para pessoas com doenças renais e pacientes em risco, devemos aumentar a conscientização desde formuladores de políticas até pacientes e a população em geral, aproveitar estratégias inovadoras para apoiar todos os quadros de profissionais de saúde e equilibrar lucros com preços razoáveis (Tabela 2). Se reduzirmos a lacuna entre o que sabemos e o que fazemos, a saúde renal se tornará uma realidade em todo o mundo.

**Tabela 2:** Exemplos de estratégias para melhorar a implementação do cuidado adequado da DRC

Domínio	Soluções em potencial
Política de saúde	Incluir as DNTs e a DRC como prioridades de saúde; garantir o financiamento sustentável; monitorar o ônus e os desfechos da doença; registros; ação multissetorial; promover a saúde renal por meio de medidas de saúde pública; alcançar os ODSs
Sistemas de saúde	Integrar o tratamento da DRC à atenção primária sob a CUS; estabelecer padrões de qualidade; incluir diagnósticos e medicamentos necessários nas listas nacionais de medicamentos/diagnósticos essenciais; monitorar e avaliar; reduzir a fuga de cérebros; monitorar a equidade; simplificar e agilizar as diretrizes
Garantia de qualidade	Regulamentação e monitoramento da qualidade dos medicamentos, especialmente dos genéricos. Monitoramento de desfechos de saúde e processos de atendimento para permitir melhorias iterativas
Profissionais de saúde	Reduzir a pressão de tempo; melhorar o conhecimento; ampliar o escopo da prática (por exemplo, farmacêuticos); envolver os agentes comunitários de saúde
Empoderamento do paciente	Alfabetização em saúde; educação; participação da comunidade; envolvimento no desenho e na condução da pesquisa
Custo de medicamentos	Genéricos de qualidade; redução de preços; CUS para medicamentos essenciais
Pesquisa de implementação	Identificar barreiras dentro dos contextos locais; testar soluções para superar as barreiras
Polipílulas	Reduzir custos; diminuir a carga de comprimidos
Tecnologias digitais	Caixas, bolsas e frascos eletrônicos de comprimidos; tecnologia de blisters; sensores ingeríveis; sistemas eletrônicos de manejo de medicamentos; tecnologia de autorrelato do paciente; tecnologia com base em vídeo; tecnologia de sensor de movimento; telemedicina; aplicativos para smartphones; prontuários eletrônicos de saúde

Abreviações – DRC: doença renal crônica; DNT: doença não transmissível; ODS: objetivo de desenvolvimento sustentável; CUS: cobertura universal de saúde.

## Divulgação

VL é presidente do *Advocacy Working Group*, Sociedade Internacional de Nefrologia, sem divulgações financeiras. KRT recebeu auxílios à pesquisa dos Institutos Nacionais de Saúde (*National Institute of Diabetes and Digestive and Kidney Diseases*, *National Heart, Lung, and Blood Institute*, *National Center for Advancing Translational Sciences*, *National Institute on Minority Health and Health Disparities*, gabinete do diretor), dos Centros de Controle e Prevenção de Doenças dos EUA e da *Travere Therapeutics*, além de honorários de consultoria da AstraZeneca, Bayer, Boehringer Ingelheim, Eli Lilly e Novo Nordisk. Ela é presidente do *Diabetic Kidney Disease Collaborative* para a Sociedade Americana de Nefrologia. RC-R é membro do Comitê Diretor do Dia Mundial do Rim, membro do Comitê de Diabetes da Sociedade Latino-Americana de Nefrologia e Hipertensão (SLANH) e membro do Conselho Regional Latino-Americano, Sociedade Internacional de Nefrologia. Ele é membro do Comitê Diretor do ensaio *Dapagliflozin and Prevention of Adverse Outcomes in Chronic Kidney Disease* (DAPA-CKD) (AstraZeneca), do *Study of Diabetic Nephropathy with Atrasentan* (SONAR) (Abbvie), do *A Non-interventional Study Providing Insights Into the Use of Finerenone in a Routine Clinical Setting* (FINE-REAL) (Bayer) e do CKD-ASI (Boehringer). Recebeu auxílios à pesquisa da AstraZeneca, GlaxoSmithKline, Roche, Boehringer e Novo Nordisk; e recebeu honorários como palestrante da AstraZeneca, Bayer, Boehringer Ingelheim e Amgen. Todos os outros autores declararam não haver interesses conflitantes.

## Material Suplementar

O seguinte material online está disponível para o presente artigo:

Figura S1 - Classificação da disfunção renal como causa de óbito estratificada por categoria de renda mundial e sexo, por fatores de risco de nível 2 para óbito.

Tabela S1 - Novos agentes terapêuticos aprovados e emergentes para diferentes doenças renais.

Tabela S2 - Comentários dos pacientes sobre acessibilidade, viabilidade econômica, conhecimento, facilitadores e obstáculos ao tratamento renal ideal.
